# Combining Acoustic Bioprinting with AI-Assisted Raman Spectroscopy for High-Throughput Identification of Bacteria in Blood

**DOI:** 10.1021/acs.nanolett.2c03015

**Published:** 2023-03-01

**Authors:** Fareeha Safir, Nhat Vu, Loza F. Tadesse, Kamyar Firouzi, Niaz Banaei, Stefanie S. Jeffrey, Amr. A. E. Saleh, Butrus (Pierre) T. Khuri-Yakub, Jennifer A. Dionne

**Affiliations:** † *Department of Mechanical Engineering, Stanford University, Stanford, California 94305, United States; ‡ Pumpkinseed Technologies, Inc., Palo Alto, California 94306, United States; § Department of Bioengineering, Stanford University School of Medicine and School of Engineering, Stanford, California 94305, United States; ∥ E. L. Ginzton Laboratory, Stanford University, Stanford, California 94305, United States; ⊥ Department of Pathology, Stanford University School of Medicine, Stanford, 94305 California, United States; # Clinical Microbiology Laboratory, Stanford Health Care, Palo Alto, California 94304, United States; ∇ Department of Infectious Diseases and Geographic Medicine, Stanford University School of Medicine, Stanford, California 94305, United States; ○ Department of Surgery, Stanford University School of Medicine, Stanford, California 94305, United States; ● Department of Engineering Mathematics and Physics, Cairo University, Cairo 12613, Egypt; □ Department of Materials Science and Engineering, Stanford University, Stanford, California 94305, United States; ■ Department of Electrical Engineering, Stanford University, Stanford, California 94305, United States; △ Department of Radiology, Molecular Imaging Program at Stanford (MIPS), Stanford University School of Medicine, Stanford, California 94035, United States

**Keywords:** acoustic bioprinting, surface-enhanced Raman spectroscopy, machine learning, infectious disease, gold nanorods, bacteria

## Abstract

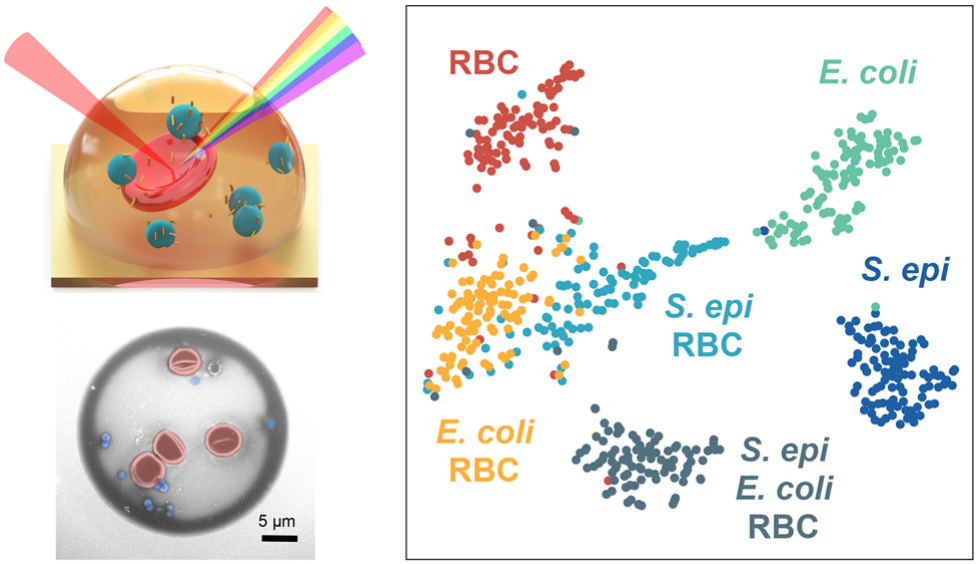

Identifying pathogens in complex samples such as blood, urine, and wastewater is critical to detect infection and inform optimal treatment. Surface-enhanced Raman spectroscopy (SERS) and machine learning (ML) can distinguish among multiple pathogen species, but processing complex fluid samples to sensitively and specifically detect pathogens remains an outstanding challenge. Here, we develop an acoustic bioprinter to digitize samples into millions of droplets, each containing just a few cells, which are identified with SERS and ML. We demonstrate rapid printing of 2 pL droplets from solutions containing *S. epidermidis*, *E. coli*, and blood; when they are mixed with gold nanorods (GNRs), SERS enhancements of up to 1500× are achieved.We then train a ML model and achieve ≥99% classification accuracy from cellularly pure samples and ≥87% accuracy from cellularly mixed samples. We also obtain ≥90% accuracy from droplets with pathogen:blood cell ratios <1. Our combined bioprinting and SERS platform could accelerate rapid, sensitive pathogen detection in clinical, environmental, and industrial settings.

Reliable detection and identification of microorganisms is crucial for medical diagnostics, environmental monitoring, food production, biodefense, biomanufacturing, and pharmaceutical development. Samples can contain anywhere from 10^6^ colony-forming units (CFU)/mL to as few as 1–100 CFU/mL.^1−3^ Though *in vitro* liquid culturing is typically used for pathogen detection, it is estimated that less than 2% of all bacteria can be readily cultured using current laboratory protocols. Further, amongst that 2%, culturing can take hours to days depending on the bacterial species.^4−7^ In the case of diagnostics, broad-spectrum antibiotics are often administered while waiting for culture results, leading to an alarming rise in antibiotic-resistant bacteria.^8^ We postulated that culture-free methods to detect pathogens in complex, multicellular samples might be possible by first digitizing samples into single- to few-cellular droplets with bioprinting, then rapidly interrogating each droplet with Raman spectroscopy, and finally classifying the results using machine learning.

Raman spectroscopy is a label-free, vibrational spectroscopic technique that has recently emerged as a promising platform for bacterial species identification.^9−12^ Since every cell species and strain has a unique molecular structure, they have a unique spectral fingerprint that can be used for identification.^10^ Compared to nucleic-acid-based tests such as the polymerase chain reaction (PCR)^13−15^ and protein-based tests such as matrix-assisted laser desorption/ionization time-of-flight mass spectrometry (MALDI-TOF)^16,17^ and enzyme-linked immunoassay (ELISA),^18,19^ Raman requires minimal to no use of reagents or labels, with relatively low-cost equipment and the potential for amplification-free detection.^20−23^ Furthermore, Raman is a nondestructive technique, with excitation laser powers low enough for living cells^24,25^ and negligible interference from water, allowing for minimal sample preparation.^26^ Combined with plasmonic or Mie-resonant nanoparticles, Raman signals can be enhanced on average by 10^5^–10^6^, and up to 10^10^,^27−29^ allowing for rapid interrogation of cells. With these advantages, Raman has been successfully applied to genetic profiling,^30^ protein detection,^31−34^ and even single-molecule detection^35−37^ (Supplementary Note 1). More recent work has also shown exciting advances in Raman for cellular identification, including bacterial identification,^10,38^ immune profiling,^39,40^ and in vivo biopsies.^41^

To advance Raman spectroscopy to clinical and industrial relevance, it must be combined with facile sample preparation methods. Nominally, the millions to billions of cells in milliliter-scale volumes found in key target samples would need to be processed within seconds to minutes. Acoustic droplet ejection (ADE) is among the most promising droplet generation platforms for biological samples. In ADE, ultrasonic waves are focused at the fluid–air interface, giving rise to radiation pressure that ejects a droplet from the surface. The diameter of the ejected droplet is inversely proportional to the frequency of the transducer, with 5 and 300 MHz ultrasonic waves generating droplet diameters of 300 and 5 μm, respectively (Supplementary Figure 1).^42,43^ Unlike other commercial piezo or thermal inkjet printers, the size, speed, and directionality of the ADE droplets are completely controlled by the sound waves without the need for a physical nozzle.^42^ As a nozzleless technology, acoustic droplet ejection has an unparalleled advantage in handling biological samples; in particular, it eliminates clogging, sample contamination, and compromised cell viability or biomarker structure due to shear forces from the nozzle. Furthermore, ADE allows for high-throughput droplet generation, processing fluids at rates of up to 25000 droplets/s or approximately 50 nL/s for a single ejector head. Microelectromechanical-system (MEMS)-based arrays of 1024 ejector heads have been previously reported, showing potential for processing volumes of over 180 mL in under 1 h^44^ as compared with the days required by existing microfluidic cell separation methods.^45^ Additionally, as this platform relies on acoustic waves, these waves can propagate through a matched coupling media with minimal loss of acoustic energy while avoiding any direct contact between the sample and the transducer. This eliminates any cross-sample contamination and maintains sterility (Supplementary Note 2).

Here, we demonstrate a novel approach for rapid pathogen identification in complex, multicellular samples by combining Raman spectroscopy with acoustic droplet ejection. We develop a bioprinter to allow sub 5 pL droplets, each consisting of a variety of cells printed with and without GNRs; thousands of droplets are printed within seconds (1 kHz rates). To our knowledge, this is the first demonstration of stable and precise high-frequency (147 MHz) acoustic printing of multicomponent samples printed from both microscale biological entities (bacterial cells and RBCs) along with nanoscale particles (GNRs). We leverage this novel liquid-sample digitization method to facilitate high-throughput SERS identification of cells within individual droplets using advanced ML classification approaches. This approach allows us to sensitively and specifically detect individual cells within a complex liquid sample and gain insights about those cells.

We print samples of mouse red blood cells, suspended in an solution of aqueous ethylenediaminetetraacetic acid (EDTA), with spike-ins of Gram-positive *Staphylococcus epidermidis* (*S. epi*) bacteria and Gram-negative *Escherichia coli* (*E. coli*), as well as gold nanorods (GNRs). Then, we collect Raman spectra from each printed droplet, using the optical signature to identify the cell constituents. We train machine-learning algorithms on samples printed from uniform cell types as well as mixed-cell samples to identify the droplet constituents. By optimizing our printing parameters, cell to nanorod concentrations, buffer solutions, and substrates, we achieve a high Raman signal across cells while correctly identifying cell types in each droplet. We achieve cellular classification accuracies of ≥99% from single-cell-line prints and ≥87% from mixed-pathogen samples, validated using scanning electron microscopy images of our droplets to confirm the presence of particular cells. Furthermore, we identify key spectral bands for classification by determining wavenumber importance and confirm that these features correspond to biologically relevant components within our known cell lines. Our work lays a foundation for future SERS-based bioprinting diagnostic platforms, paving the way for rapid, specific, sensitive, label-free, and amplification-free detection of live cells.

We built a zinc oxide 147 MHz transducer bonded to a quartz focusing lens with a focal distance of 3.5 mm. The transducer is encased in a stainless steel housing and mounted 3.5 mm above a machined stainless steel plate with a 1 mm diameter hole through which droplets are ejected downward (Supplementary Figure 2a). 200 μL of sample solution is pipetted between the transducer and this plate to fill the 3.5 mm focal distance of the transducer. The aperture is large enough to negate any nozzlelike effects, and the fluid is held in place against the transducer and the plate through surface tension (Figure 1a). We position a motorized, programmable *xy* stage 1 mm beneath this plate, allowing for patterned ejection. The setup is monitored through a stroboscopic camera mounted opposite to an LED to evaluate droplet stability and ejection (Figure 1b and Supplementary Figures 2b, 3a,b, and 4). After first experimenting with a range of frequencies and droplet diameters (Figure 1c and Supplementary Figure 1), we selected our 147 MHz transducer frequency with droplet diameters of ∼15 μm or ∼2.15 pL in volume, to match the order of magnitude of our cellular diameters. We found that this volume allows us to print droplets with a number of cells in each droplet, while also maximizing Raman enhancement from GNR coating.^46,47^

**Figure 1:**
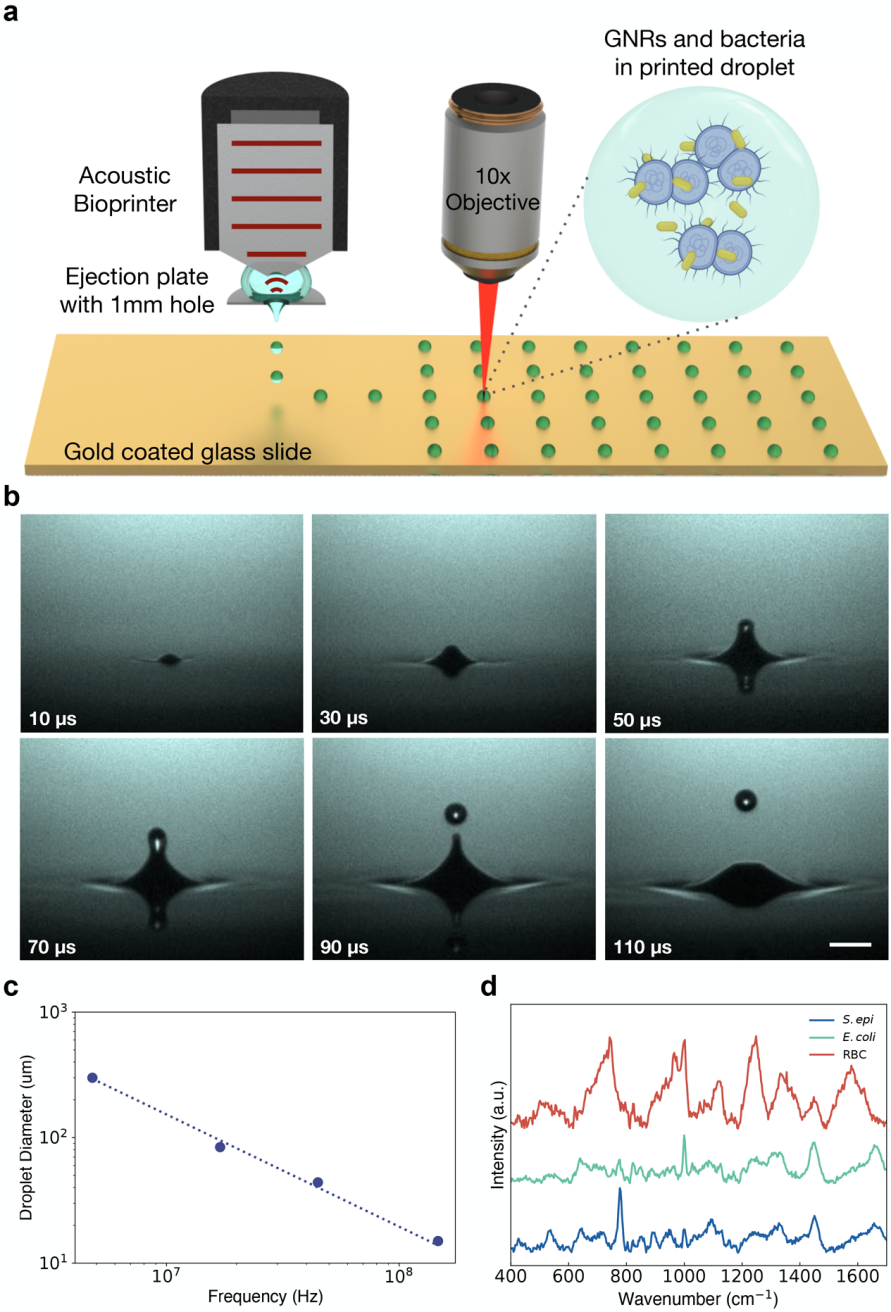
(a) Schematic of acoustic printing platform and confocal Raman setup. Droplets containing bacteria (purple) and nanorods (gold) suspended in EDTA solution are acoustically printed onto a glass slide coated in 200 nm of gold (see also Supplementary Figures 2–4). (b) Stroboscopic images of the time evolution of upward droplet ejection at ∼3.5 m/s from an open pool at an acoustic frequency of 44.75 MHz and a droplet ejection repetition rate of 1 kHz. Images were captured with an exposure time of 40 ms, and as such, each frame is composed of 40 droplet ejections, highlighting ejection stability. The scale bar is 100 μm (see also Supplementary Figure 2). (c) Graph of droplet diameter versus ultrasound transducer resonant frequency. Droplets were printed with 4.8, 17, 44.75, and 147 MHz and had droplet diameters of 300, 84, 44, and 15 μm, respectively, highlighting the tunability of acoustic droplet ejection. (see also Supplementary Figure 1). (d) Raman spectra of dried cellular samples, including *S. epi*, *E. coli*, and red blood cells (RBCs) on a gold-coated slide.

We synthesized GNRs with a longitudinal plasmon resonance of 960 nm, chosen to be used with an excitation wavelength of 785 nm to minimize background fluorescence. We synthesized GNRs to be close enough to our laser line to be excited by our laser but red-shifted enough to minimize competitive extinction of the incident and Raman-scattered light^38,48,49^ (Figure 1d and Supplementary Figures 5 and 6a,b). UV–vis absorption spectra and transmission and scanning electron micrographs (TEM and SEM) of the gold nanorod samples confirm the strong near-infrared plasmon resonance peak and reasonable sample monodispersity (Supplementary Figure 5). All rods were coated in sodium oleate and hexadecyl(trimethyl)ammonium bromide (CTAB), which gives them a slight positive charge,^38^ further increasing binding with our negatively charged bacteria^38,50^ and, to a lesser degree, the negatively charged RBCs.^51,52^

For this study, cells were suspended in a 1:9 volumetric mixture of EDTA and deionized water, diluted to a final concentration of 1e9 cells/mL. This solution was chosen to prevent hemolysis of our red blood cells (RBCs), while avoiding crystallization upon drying present in droplets printed from salt-based buffers (Supplementary Figure 7). Furthermore, we have observed that the inclusion of EDTA provides a denser coating of GNRs on cell surfaces with few rods located elsewhere in the droplet (Supplementary Figure 8);^53^ we hypothesize such a coating is due to the interaction between the surface charge of our cells and the CTAB on the GNRs. Samples were printed on silane-treated, gold-coated glass substrates to minimize background spectra in the region of interest while further inducing coating of GNRs on our cells through their hydrophobicity (Supplementary Figures 9 and 10).

We tune the acoustic pulse width and input power into our transducer and ensure our printer is in focus for each sample to ensure that we can reliably and precisely print patterned grids of droplets containing bacteria and RBCs with GNRs and without GNRs, printed at ejection rates of 1 kHz, as shown in Figure 2a. Grid prints of additional cell line mixtures can be found in Supplementary Figure 11. Furthermore, we maintain cell viability during printing, as demonstrated by the positive growth of cells printed directly onto agar-coated slides. Figure 2b, for example, shows droplets of *E. coli* bacteria grown 0, 12, 24, and 36 h postprinting, demonstrating the maintained viability of the cells after acoustic droplet ejection.

**Figure 2:**
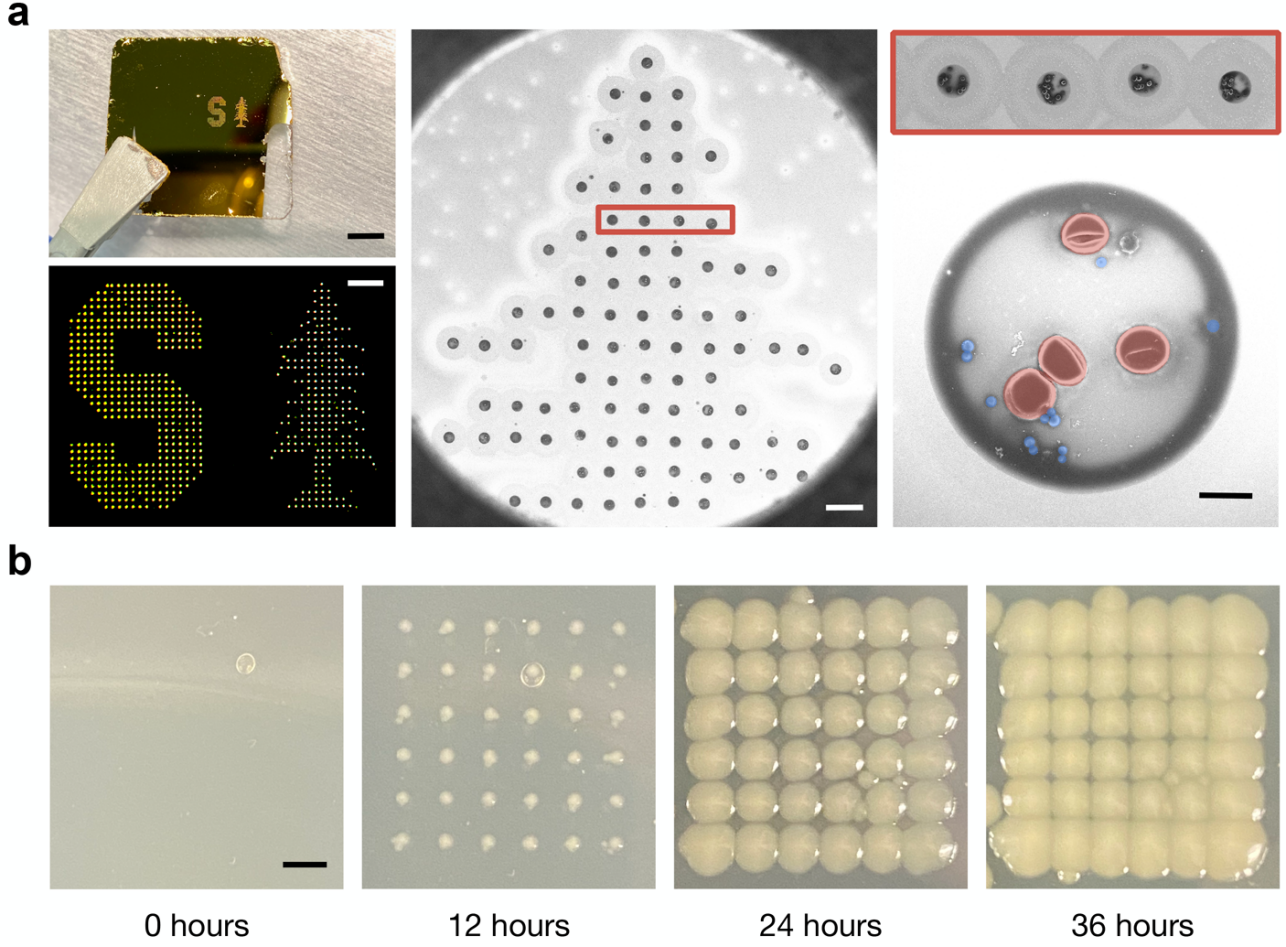
Patterned droplet ejection from cellular stock solution. All droplets were ejected at 147 MHz. (a) Pattern printout of the Stanford University logo printed from droplets containing a 1:1 mixture of *S. epi* bacteria and mouse RBCs onto a gold-coated slide. The image on the left shows a photograph of print (top) with a scale bar of 4 mm. The brightfield image (bottom) was collected using a 5× objective lens and has a scale bar of 500 μm. (middle) SEM of the top portion of the tree region of the print with a scale bar of 100 μm. (right) A single row of 4 droplets from the large-area print, and then a magnified image of a single droplet with false coloring showing RBCs in red and *S. epi* bacteria in blue. The scale bar is 5 μm. (b) Droplets containing *E. coli* bacteria were printed onto an agar-coated slide and incubated at 37 °C for up to 36 h to demonstrate the cellular viability of printed samples. 100 droplets were placed at each location to ensure each droplet contained cells. The scale bar is 2 mm.

SERS spectra from our acoustically printed droplets are collected using a 785 nm laser (Supplementary Figure 12). We first print grids of droplets from 6 cellularly pure samples: *S. epi*, *S. epi* with GNRs, *E. coli*, *E. coli* with GNRs, mouse RBCs, and mouse RBCs with GNRs (Figure 3a and Supplementary Figure 11). Figure 3b shows a magnified SEM of the droplet printed with *S. epi* and GNRs and demonstrates that our cells are abundantly coated with GNRs. The normalized, average signal from 100 droplets of each cellular sample with GNRs and average signal from 15 droplets of each sample without GNRs are shown in Figure 3c, with spectral acquisition times of 15 s for each droplet (Supplementary Figures 13–16). Note that little to no signal is observed with this collection for droplets without the nanorods. Relative signal intensities for non-normalized samples with data standard deviations can be found in Supplementary Figure 17. While our work on cellular identification was performed using isolated RBCs, we demonstrate our platform’s ability to work on more complex samples by precisely printing droplets from mouse whole blood diluted with an anticoagulant (EDTA) and mixed with GNRs, without the need for any further sample preprocessing. We then collect Raman spectra from these droplets and show that we maintain the spectral peaks found in our pure RBC sample with the presence of additional peaks as would be expected of this more complex sample. Spectra and SEMs can be found in Supplementary Figure 18.

**Figure 3:**
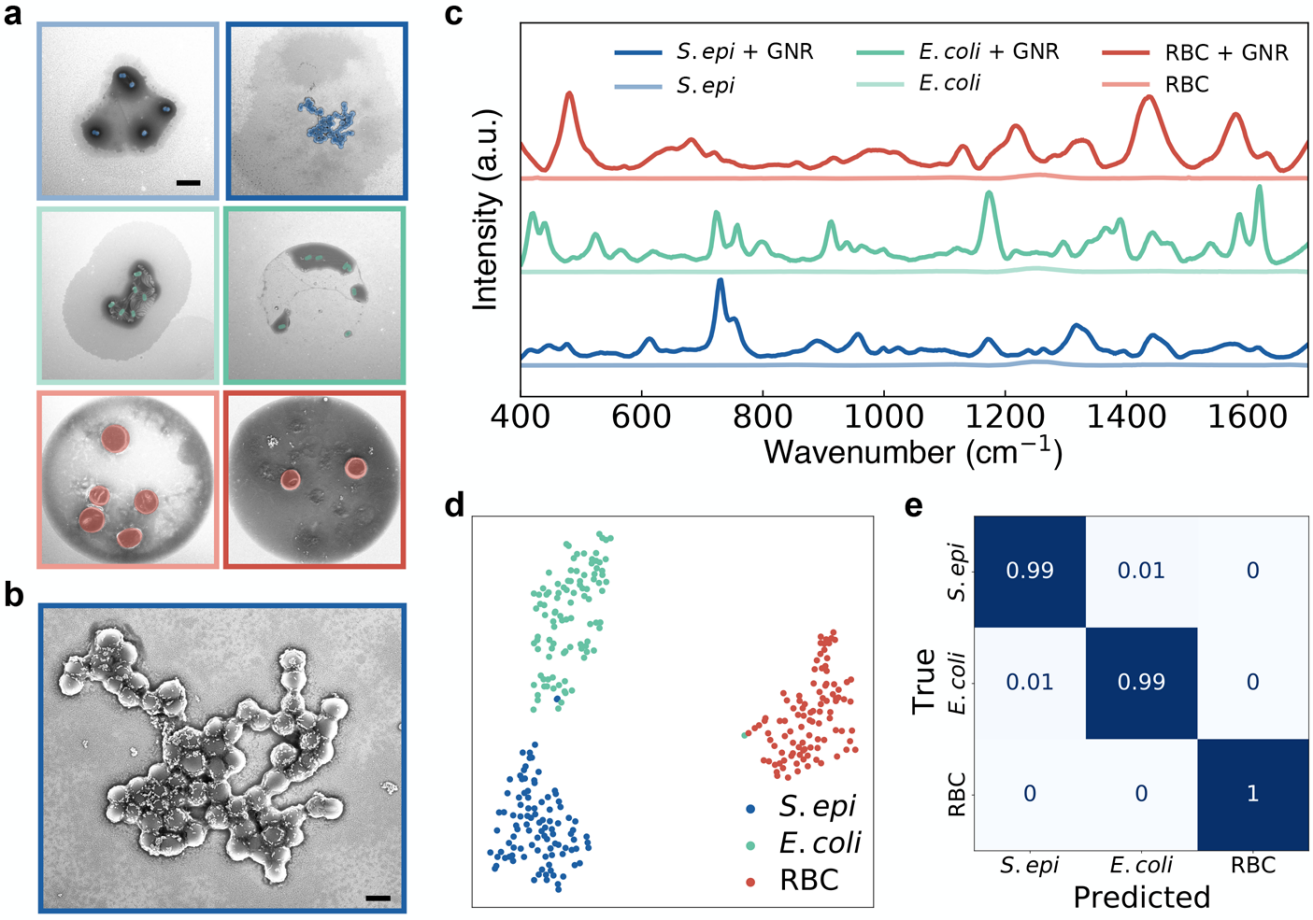
Spectral identification of cells printed with GNRs. (a) SEMs showing single droplets printed from varying cellular samples suspended in our EDTA solution at a concentration of 1e9 cells/mL. The left column shows samples without GNRs, and the right column shows cells printed with GNRs. From top to bottom, droplets contain *S. epi*, *E. coli*, and RBCs with false coloring added to highlight the cells. The scale bar is 5 μm. (b) Magnified SEM of a droplet containing *S. epi* coated with GNRs from (a). SEM highlights that the bacteria are coated with GNRs with very few rods dispersed in the rest of the droplet. The scale bar is 2 μm. (c) Mean SERS spectra of 100 measurements each taken from single droplets printed from three cell lines (*S. epi*, *E. coli*, and RBCs) mixed with GNRs. (d) 2-component t-SNE projection across all 300 Raman spectra acquired from droplets printed with GNRs. Data is plotted after performing a 24-component PCA for dimensionality reduction. Plots show distinct clustering of our cell lines. (e) Normalized confusion matrix generated using a random forest classifier on the 300 spectra collected from single cell-line droplets of *S. epi*, *E. coli*, and mouse RBCs mixed with GNRs. Samples were evaluated by performing a stratified *K*-fold cross-validation of our classifier’s performance across 10 splits, showing ≥99% classification accuracy across all samples.

Our data show significant Raman signal enhancement from the sample sets with nanorods compared to the controls, estimated at 300–1500×. For a more precise classification of our droplet mixtures, we start by reducing the dimensionality of our spectra from 508 wavenumbers to 24 components using PCA in order to prevent classifier oversampling due to our data set having more features than samples. We show that the first 24 principal components account for >90% of our sample variance (Supplementary Figure 19), and we still see clear sample differentiation between each data set and cell type on a 2-component *t*-distributed stochastic neighbor embedding (t-SNE) projection after PCA (Figure 3d and Supplementary Figure 20). We then use a random forest classifier for our multiclass analysis from our complex samples. We tune our classifier hyperparameters using a cross-validated grid search to generate optimized parameters. Inputting these parameters into our classifier, we take 100 spectra from each of our 3 classes of cellular samples with GNRs and perform a stratified *K*-fold cross-validation of our classifier’s performance across 10 splits and demonstrate ≥99% classification accuracy across all samples (Figure 3e).

We demonstrate that we can accurately classify droplets printed at 147 MHz from complex, clinically relevant cellular mixtures. We print arrays of droplets from 200 μL of solution formed from equal mixtures of *S. epi* and RBCs, *E. coli* and RBCs, and *S. epi*, *E. coli*, and RBCs, all diluted to a final concentration of 1e9 cells/mL of each cell type in our aqueous EDTA solution and mixed with GNRs (Figure 4a). We collect single-droplet SERS spectra from our mixture printouts, identically to that of our single cell-line droplets, using a 785 nm laser with a 15 s acquisition time. We then evaluate 100 spectra each of all 6 classes of our samples, the 3 single-cellular samples presented in Figure 3 and our 3 mixture classes. We reduce the dimensionality of our samples to 30 components using PCA, sufficient to account for >90% of our sample variance (Supplementary Figure 21) and plot a 2-component t-SNE projection to show clear clustering between each data set (Figure 4b and Supplementary Figure 22). We then retune our classifier hyperparameters with our new data, evaluate our samples using a random forest classifier with a stratified *K*-fold cross-validation as previously described, and demonstrate ≥87% classification accuracy across all samples (Figure 4c).

**Figure 4:**
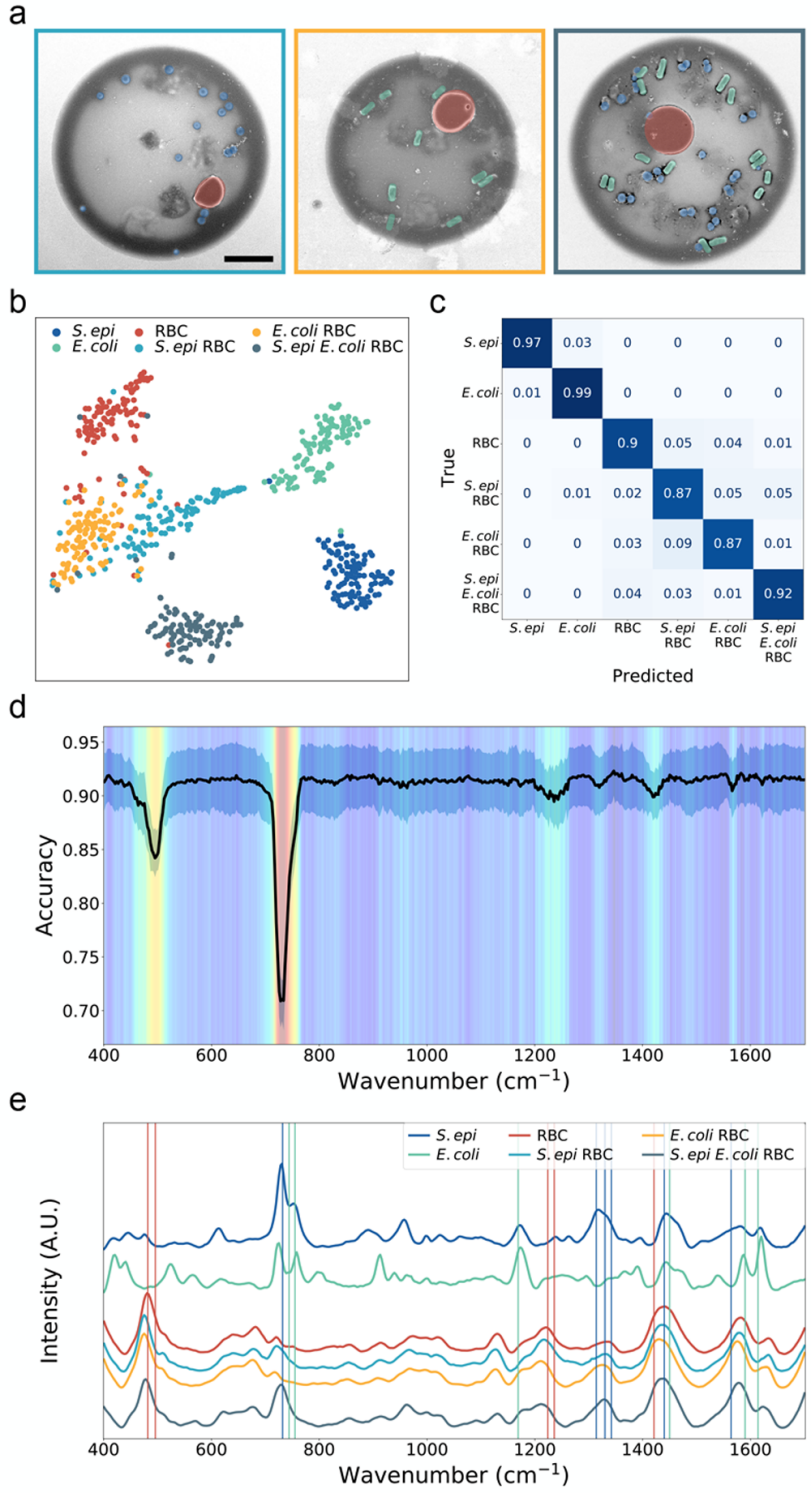
(a) False-color SEMs of droplets printed from (left to right) an equal mixture of *S. epi* bacteria and RBCs, *E. coli* bacteria and RBCs, and *S. epi*, *E. coli*, and RBCs all diluted to 1e9 cells/mL in aqueous EDTA and mixed with GNRs. The scale bar is 5 μm. (b) 2-component t-SNE projection across all 600 Raman spectra acquired from 100 droplet measurements each, taken from single droplets printed from three cell lines (*S. epi*, *E. coli*, and RBCs) and three mixtures (*S. epi* and RBCs, *E. coli* and RBCs, and *S. epi*, *E. coli*, and RBCs) mixed with GNRs. Data are plotted after performing a 30-component PCA for dimensionality reduction. Plots show clustering of our cell lines with the most overlap between droplet mixture samples. (c) Normalized confusion matrix generated using a random forest classifier on the 600 spectra collected from single-cell-line droplets of *S. epi*, *E. coli*, and mouse RBCs mixed with GNRs and our 3 cell mixtures. Samples were evaluated by performing a stratified *K*-fold cross-validation of our classifier’s performance across 10 splits, showing ≥87% classification accuracy across all samples. (d) Heat map highlighting feature extraction performed to determine the relative weight of spectral wavenumbers in our random forest classification. The heat map is overlaid with a plot of the mean and standard deviation of the classification accuracy (black) calculated across all trials. Wavenumbers with lower accuracies are shown to be critical features, as random perturbations are highly correlated with decreases in classification accuracy. (e) Plots of the mean SERS spectra of 100 measurements each, taken from single droplets printed from three cell lines (*S. epi*, *E. coli*, and RBCs) and three mixtures (*S. epi* and RBCs, *E. coli* and RBCs, and *S. epi*, *E. coli*, and RBCs) mixed with GNRs. Wavenumbers attributed to biological peaks found in SERS spectra of *S. epi*, *E. coli*, and RBCs are plotted as blue, green, and red vertical lines, respectively. Peak assignments can be found in Supplementary Table 1.

To verify that our classifier is using physiologically meaningful spectral bands for prediction, we compute the feature importance at each wavenumber and validate that high-importance bands correspond to specific biological components and vibrations in our cells. To identify these meaningful bands, we start by repeatedly splitting our 600 spectra into random 80/20 train/test splits and train a model on each training set. For the test set, we iterate through the wavenumbers and, at each iteration, perturb the spectrum by modulating the amplitude with a Voigt distribution. After each perturbation, we recalculate the classification accuracy, compare the updated results with our baseline accuracy, and determine the importance for each wavenumber—the greater the decrease in accuracy due to our perturbation, the more important the wavenumber. We split our samples using a stratified shuffle split and repeat 10 times. Each wavenumber of each spectrum in the test set is perturbed 5 times, and all results are averaged to determine our final feature importance. We plot a heat map highlighting the relative wavenumber importance overlaid with a plot of the mean and standard deviation of the perturbed classification accuracy (Figure 4d and Supplementary Figures 23 and 24). We further plot the normalized, average signal from 100 droplets of each cellular sample with GNRs. Relative signal intensities for non-normalized samples with data standard deviations can be found in Supplementary Figure 25. We note that the key spectral bands highlighted by our algorithm match peaks in our spectra and that these distinct peak wavenumbers represent bands previously reported in the literature of dried and liquid SERS of our cell lines, including *S. epi*, *E. coli*, and RBCs (Figure 4e and Supplementary Figure 26).^38,54−64^ We specifically note that peaks at 732.5 and 1330 cm^–1^ from our *S. epi* containing samples are attributed to purine ring-breathing modes^59^ and the adenine part of the flavin derivatives or glycosidic ring mode of polysaccharides,^58^ peaks at 755 and 1450 cm^–1^ from our *E. coli* containing samples are attributed to tryptophan ring breathing^64^ and CH_2_/CH_3_ deformation of proteins and lipids,^56^ and peaks at 482 and 1224 cm^–1^ from our RBC-containing samples are attributed to the γ12 out of plane deformation of porphyrin, a main component of hemoglobin,^61^ and ν13 or ν42 valence.^62^ Further peak assignments can be found in Supplementary Table 1.

While our Raman analysis was on samples printed with high concentrations of blood and bacteria, much lower cell concentrations should be detectable. For example, we can analyze droplets that contain a smaller number of *S. epi* bacteria than RBCs. As shown in Supplementary Figure 27, droplets that have pathogen:RBC ratios of less than 1 still exhibit high classification accuracies of 90%. Therefore, we can detect a bacterial signal from a printed droplet even with low cell counts. Furthermore, Raman interrogation can also be performed within each droplet. As shown in Supplementary Figures 28 and 29, we collect spectral maps across a single droplet. Even in these mappings, our ML classifier can identify the cell type of each individual spectrum and predict the cellular makeup of each droplet. Additionally, we demonstrate that our machine-learning algorithm for wavenumber importance can determine relevant feature bands by individual sample classes. We show that different bands carry differing weights for the classification of each sample class, with the largest differences being present between bacterial and blood cells. With these results, we propose a vision for rapid, hyperspectral Raman imaging that allows for bacterial identification without the need for full spectroscopic analysis of each droplet. We propose to separately image an array of printed droplets at the bands that have the greatest feature importance for blood and bacteria, respectively. Droplets containing only red blood cells would “light up” or have high intensity at one wavelength, while those few droplets containing a mixture of bacteria and red blood cells would “light up” at a different wavelength, allowing for rapid identification of droplets containing bacteria using this hyperspectral Raman imaging technique (Supplementary Figure 30).

We have demonstrated a rapid platform for acoustic-printing-based droplet SERS of biological samples. Our system enables rapid digitization of cells from fluid samples in picoliter droplets with minimal sample contamination through nozzle-free acoustic printing at kilohertz ejection rates. As a result of our choice of printer frequency, cell stock solution, and slide surface treatment, our platform generates droplets containing cells uniformly coated in GNRs. Our results show that we can stably print samples of cells with and without GNRs and can demonstrate clear signal enhancements of up to 1500× from the addition of our GNRs. Furthermore, from these droplets, we demonstrate single-droplet Raman interrogation and cellular identification in 15 s. We show that we generate these consistent Raman spectra from Gram-positive and Gram-negative bacteria as well as from RBCs and can differentiate spectra. Finally, we demonstrate that we can identify distinct cell types present in droplets printed from a mixture of cell lines using machine-learning algorithms.

Our work could advance Raman-based clinical research, clinical diagnostics, and disease management. Minimally invasive, fluid-based biomarker detection is gaining traction for the development of new point-of-care systems. A reliable and automated biological acoustic printer coupled with SERS nanoparticles and Raman hyperspectral imaging could be used to separate, count, and identify various cell lines, allowing for rapid, specific, and label-free cellular analysis. Furthemore, ADE-based SERS could be designed with an array of ejector heads to rapidly split large patient sample volumes, or a single ejector could provide detailed analysis of a small volume, minimizing the use of expensive reagents. As such, ADE-based SERS could enable culture-free cellular identification and monitoring from samples with low concentrations or from samples with species that are difficult to culture, including circulating tumor cells (CTCs) for cancer screening and monitoring,^65−67^ CD4 levels for HIV monitoring,^65,68^ and strain-specific identification of slow-growing *Mycobacterium tuberculosis* for treatment planning.^69−71^ Additionally, given that acoustic printing is nozzle-free and contactless, ADE-based SERS could facilitate easy multiplexing of various patient samples or other relevant media, as the ejector can easily scan across a number of different sample wells without risking contamination. Lastly, given the versatility of our substrates, colloidal GNRs, and printing platform, our system is not limited to processing cells but could easily be modified for use in detecting other biomarkers including small molecules and proteins, coupled with surface chemistry for labeled detection of nucleic acids, and used for low-volume interrogation of pharmaceutical samples in drug development. Our work in integrating SERS cellular interrogation with acoustic bioprinting and machine learning provides a foundation for further research into rapid, cellular-based diagnostics and paves the way for reliable, low-cost point-of-care diagnostics.

## Supporting Information Available

Materials and methods, gold nanorods for SERS applications, acoustic printing for handling biological samples, supplementary schematics of experimental setup, gold nanorod characterization, Raman spectra of control samples, mean and standard deviation of acquired Raman spectra, Raman spectra of mouse whole blood samples, t-SNE plots of data before and after PCA dimensionality reduction, feature validation, classification of samples with a greater number of red blood cells than bacteria, intradroplet Raman mapping, proposed vision for Raman hyperspectral imaging, plot of spectral preprocessing, and tentative band assignments of the SERS spectra of *S. epi*, *E. coli*, and RBCs as reported in the literature (PDF)
