# Supplementary material for: Combining Acoustic Bioprinting with AI-Assisted Raman Spectroscopy for High-Throughput Identification of Bacteria in Blood
Source: Nano Lett. 2023 Mar 1;23(6):2065–73. doi: 10.1021/acs.nanolett.2c03015 (PMC10037319; doi:10.1021/acs.nanolett.2c03015)
Supplement: Supplementary file 1 — nl2c03015_si_001.pdf [file nl2c03015_si_001.pdf]

Supplementary Information: Combining acoustic bioprinting with AI-assisted Raman spectroscopy for high-throughput identification of bacteria in blood.

Fareeha Safir[1*], Nhat Vu[2], Loza F. Tadesse[3], Kamyar Firouzi[4], Niaz Banaei[5,6,7], Stefanie S. Jeffrey[8], Amr. A. E. Saleh[9,10*], Butrus (Pierre) Khuri-Yakub[4,11] and Jennifer A. Dionne[10,12*]

[1*]Department of Mechanical Engineering, Stanford University, Stanford, 94305, CA, United States.
[2]Pumpkinseed Technologies, Inc., Palo Alto, 94306, CA, United States.
[3]Department of Bioengineering, Stanford University School of Medicine and School of Engineering, Stanford, 94305, CA, United States.
Present Address: Department of Electrical Engineering and Computer Science, University of California, Berkeley, Berkeley, 94720, CA, United States.
[4]E. L. Ginzton Laboratory, Stanford University, Stanford, 94305, CA, United States.
[5]Department of Pathology, Stanford University School of Medicine, Stanford, 94305, CA, United States.
[6]Clinical Microbiology Laboratory, Stanford Health Care, Palo Alto, 94304, CA, United States.
[7]Department of Infectious Diseases and Geographic Medicine, Stanford University School of Medicine, Stanford, 94305, CA, United States.
[8]Department of Surgery, Stanford University School of Medicine, Stanford, 94305, CA, United States.
[9]Department of Engineering Mathematics and Physics, Cairo University, Cairo, 12613, Egypt.
[10]Department of Materials Science and Engineering, Stanford University, Stanford, 94305, CA, United States.
[11]Department of Electrical Engineering, Stanford University, Stanford, 94305, CA, United States.
[12]Department of Radiology, Molecular Imaging Program at Stanford (MIPS), Stanford University School of Medicine, Stanford, 94035, CA, United States.

*To whom correspondence should be addressed; E-mail: fsafir@stanford.edu; aessawi@eng.cu.edu.eg; jdionne@stanford.edu;

Methods

Gold nanorod synthesis and characterization

Hexadecyl(trimethyl)ammonium bromide (CTAB) and sodium oleate (NAOL) coated gold nanorods were synthesized following previously described protocols[1]. The nanorods were cleaned by centrifuging 1.5 mL aliquots twice at (9000 rpm, 20 min), allowing for one wash after synthesis as this has been shown to be adequate to maintain cell viability while preventing nanorod aggregation[2]. Samples were concentrated down to 10 µL to be mixed with cell samples and diluted to a final volume of 200 μL. Absorption spectra were recorded using a Cary 5000 UV-vis-NIR spectrometer. Scanning electron microscopy images were taken using FEI Magellan 400 XHR Scanning Electron Microscope (SEM). Transmission electron microscopy images were taken using FEI Tecnai G2 F20 X-TWIN Transmission Electron Microscope (TEM).

Scanning electron microscopy (SEM) of printed samples

For scanning electron microscope (SEM) imaging, printed samples were imaged after completion of all Raman Spectroscopy. Samples were prepared by evaporating a $\sim$10 nm layer of 60:40 gold to palladium to allow for better visualization of cells under electron beam illumination. SEM images were taken using FEI Magellan 400 XHR Scanning Electron Microscope.

Bacteria culturing and preparation

E. coli, ATCC 25922, and *S. epidermidis*, ATCC 12228, were grown from frozen stocks on Trypticase Soy Agar 5% Sheep Blood 221239 BD plates. A single colony was seeded in 10 mL Lysogeny broth (LB) culture medium and incubated at 37°C shaking at 300 rpm for 15 hrs using Thermo Scientific MaxQ 4450 incubator. 1.5 mL of culture was washed with water three times at 6000 rpm for 3 min using a mySPINTM 6 Mini Centrifuge. Samples were then concentrated down to 100 µL volumes. The cell count was collected using a Bright-Line Hemacytometer using a 1:5000 dilution of the cell culture stock solution. Stock solutions contained on average $\sim$1e10 cells/mL.

Preparation of red blood cell solutions

For data collected with purified mouse red blood cells: CD-1 (1CR) purified Mouse Red Blood Cells (RBCs) K2EDTA Gender Unspecified Pooled samples MSE00RBK2-0104095, were purchased from BioIVT in 5mL volumes. RBCs were diluted in a 1:9 v/v mixture of Invitrogen UltraPure 0.5 M EDTA, Invitrogen 15575020, to a final dilution of 1:5000 and cell counts were collected using a Nexcelom Cellometer X2 cell counter.

For data collected with mouse whole blood: CD-1 (1CR) Mouse Whole Blood K2EDTA Gender Unspecified Pooled samples MSE00WBK2-0000627, were purchased from BioIVT in 5mL volumes. The whole blood were diluted in a 1:9 v/v mixture of Invitrogen UltraPure 0.5 M EDTA, Invitrogen 15575020,

to a final dilution of 1:5000 and cell counts were collected using a Bright-Line Hemacytometer. Whole blood contained ∼1e10 cells/mL RBCs.

Preparation of mixtures for printing

Printing was completed using 200 μL of solution. All samples were diluted to a final volume of 200 μL in a 1:9 v/v mixture of Invitrogen UltraPure 0.5 M EDTA, Invitrogen 15575020, and Millipore water, unless otherwise noted. For samples with cells and no nanorods, a single concentrated cell solution or a mixture of cell solutions was diluted in aqueous EDTA to a final concentration of 1e9 cells/mL of each cell type in a given mixture. This concentration was chosen to ensure a majority of printed droplets contained at least 1 cell. For samples of cells mixed with nanorods, cleaned, concentrated nanorod solution was first mixed with concentrated cell stock solution, for our final concentration of 1e9 cells/mL per cell type, and then subsequently diluted with our aqueous EDTA solution. All solutions are mixed by inverting our microcentrifuge tubes a minimum of 10 times.

EDTA was chosen as our sample buffer to avoid crystallization upon drying seen with PBS(Supplementary Fig. 6). On top of that, EDTA provides two further advantages for our printed samples. When EDTA-containing droplets dry on a hydrophobic substrate, a central region of aggregated EDTA, cells, and GNRs dries in a much smaller area than that of a full droplet, forcing the cells and GNRs into a much smaller volume, ensuring better coverage of the cells with GNRs (Supplementary Fig. 9). Furthermore, we hypothesize that the EDTA induces aggregation among the GNRs due to the electrostatic interaction between any residual CTAB on our GNRs and the EDTA[3], as demonstrated in Supplementary Fig. 7. We hypothesize that this clustering, when coupled with the addition of cells, allowed for greater quantities of nanorods to coat the cells, and led to the creation of SERS "hot spots" amongst the aggregated GNRs, providing strong enhancements[4, 5].

Finally, we show that the addition of the EDTA and nanorods adds minimal Raman background noise (Supplementary Fig. 8, 9, 14), making it appropriate for our work in Raman cellular identification. Finally, to further minimize coffee-ring effects from nanorods upon droplet drying, we used vapor deposition to coat our gold-coated slides with a hydrophobic silane layer (3-Aminopropyl)triethoxysilane (APTMS) which allows for a more close packing of our GNRs, providing greater and more uniform enhancement on our cells[6–8].

Fabrication of silanized, gold-coated glass slides

The gold substrates used in this work were prepared by evaporating a 5 nm adhesion layer of titanium, followed by 200 nm of gold at a rate of 1 Angstrom/second using a KJ LEsker e-beam evaporator onto piranha cleaned borosilicate glass slides. The gold-coated glass slides were then cleaned with an oxygen plasma, using a Diener Pico Oxygen Plasma Cleaner, for

3 min at 100 W power and ∼2 mbar of pressure, and silanized with 3-(aminopropyl)trimethoxysilane, APTMS, using vapor deposition in order to make the surface more hydrophobic and allow for greater aggregation of the gold nanorods on the cells[8–10]. Slides were placed in a 1 liter flask in the presence of 100 µL of APTMS, Sigma-Aldrich 281778-5ML. The flask was then placed in a water bath at 40°C and allowed to react for 1 hr, after which the slides were removed from the flask and placed on a hot plate heated to 40°C for 10 min to allow for the evaporation of loosely bound molecules. We demonstrate that this APTMS layer also provides minimal Raman background noise, making it a great candidate for quick and easy substrate modification for biological Raman analysis (Supplementary Fig. 8).

Acoustic printing

Acoustic printing was completed using our custom-built ultrasonic, immersion transducer with a center frequency of 147 MHz and a focal distance of 3.5 mm (unless otherwise noted) as determined using a network analyzer, Hewlett Packard 8751A, and through pulse echo measurements taken on an oscilloscope, Keysight InfiniiVision DSOX3054A. The transducer was bound to a quartz, spherical focusing lens. The transducer was mounted on x,y,z manual translation stages, facing downwards, held 3.5 mm above a 303 stainless steel ejection plate with a 1 mm hole. For printing experiments, fluid was pipetted into the gap between the tip of the focusing lens and the ejection plate, held in place through surface tension. During printing experiments, droplets were ejected downwards through this 1 mm hole onto our chosen substrates (Supplementary Fig. 2).

To generate our droplets, our transducer was powered by a waveform generator, Keysight 33600A Series Trueform Waveform Generator. The waveform generator was connected to a synthesized RF signal generator, Fluke 6062A, which in turn is connected to a power amplifier, Minicircuits ZHL-03-5WF+. Our waveform generator produces a square-wave burst with a repetition frequency of 1 kHz, when operating continuously, at our desired pulse width of 5.5 μs and at a voltage of 1.5 volts, enough to trigger our RF synthesizer. The RF synthesizer generates a sinusoidal wave at 147 MHz and at our desired voltage, which then gets amplified before reaching the transducer. Droplets printed from deionized water were ejected with 0.096 μJ of energy, droplets printed from samples of *S. epi* and *E. coli* with and without GNRs were printed with 0.139 μJ of energy, and droplets printed with RBCs with and without GNRs and from mixtures of RBCs, *S. epi*, *E. coli*, and GNRs were all printed with 0.386 μJ of energy.

To ensure stable ejection, we monitored ejection using a camera, Allied Vision Guppy Pro F-125 1/3" CCD Monochrome Camera, coupled with a 20x objective pointed at the bottom of our ejection plate. This camera was mounted opposite a strobing LED, also triggered by our waveform generator. We also monitored the acoustic echo using an inline oscilloscope, Keysight InfiniiVision DSOX3054A. To set up our printer, we pipette in 200 µL of fluid,

turn on power to our transducer, and ensure that the transducer is in focus by manually adjusting the focal distance of our transducer until we maximize the echo as observed on the oscilloscope. We then vary the output voltage of the RF synthesizer until we stably eject a single droplet without any additional satellite droplets, as observed through our camera feed. We were then ready to pattern print arrays of droplets (Supplementary Fig. 3, 4).

Pattern printing

Pattern printing was completed using a custom 3D printed substrate holder mounted to two perpendicularly stacked Thorlabs DDS100M 100mm brushless DC linear translation stages controlled by two Thorlabs K-Cube brushless DC servo drivers. Our substrate is mounted ∼1 mm below our ejection plate to minimize droplet translation before it reaches the substrate. A MATLAB, Mathworks, 2018b, script was used to pattern print droplets onto our substrate by controlling both our motorized stages and our waveform generator to trigger droplet ejection at specific substrate locations.

Raman spectroscopy

Raman spectra was collected using the Horiba XploRa confocal Raman microscope. The excitation wavelength for all measurements was 785 nm. The Raman shift from 400 cm^{-1} to 2000 cm^{-1} was collected using 600 gr/mm grating. For baseline Raman spectra shown in Fig. 1, laser light was directed to and Raman scattered light was collected from the sample using a 100x LWD, 0.6 NA objective with spot size of 0.83 μm, with laser power at the sample of ∼6.71 mW, and acquisition time of 180 s. For spectra collected from each entire droplet, laser light was directed to and Raman scattered light was collected from the sample using a 10x, 0.25 NA objective with spot size of 2 μm, with laser power at the sample of ∼10.6 mW, and acquisition time of 15 s (Supplementary Fig. 11). Bacterial NR mixtures were measured within ∼2 hr of sample preparation.

Spectral data processing

Python (Jupyter Notebook) was used to process spectral data. For spectra preprocessing, samples were first thresholded to a minimum intensity of 150 a.u. to remove any spectra with weak signal that likely were collected on the substrate without the presence of cells. We then transformed our data by taking $\log_{10}$(y)[11, 12] and smoothed the spectra using wavelet denoising[13, 14]. To perform our smoothing, we used the *denoise_wavelet* function from the scikit-image Python library: denoise_wavelet(y, method='BayesShrink', mode='soft', wavelet_levels=1, wavelet='coif3', rescale_sigma='True'). We then performed a baseline removal by using a polynomial fit with degree 10. The specific package used and code line is: peakutils.baseline(y, deg=10, max_it =1000, tol=0.0001). Note, the need for a higher degree polynomial arises from a typical instrumental background that is difficult to fit with lower degree fits.

Following this baseline correction, Spectra were then individually normalized across all wavenumbers by subtracting the spectral mean and dividing by the standard deviation using the NumPy Python library[15], where y is the array of intensity values across all wavenumbers for each spectra: (y - numpy.mean(y))/numpy.std(y) (Supplementary Fig. 31).

For classification of samples, we further pre-process data by reducing dimensionality of our spectra from 508 to the number of components necessary to account for 90% of our sample variance using the PCA algorithm from Scikit-learn[16] (Supplementary Fig. 19, 21). Classification was performed using a Random Forest Classifier. We first tuned our classifier hyperparameters using a cross-validated grid search to generate optimized parameters. To do this we use Scikit-learn *StratifiedShuffleSplit*[16] function to randomly split our sample 20 times into an 80:20 train:test split and created a parameter grid for our number of estimators: {50, 100, 150, 200, 250, 300}, max features: {auto, sqrt, log2}, and max depth: {2, 5, 10, 15, 20}. We created our Random Forest Classifier using Scikit-learn, with a min_samples_split=2, and then performed our grid search using Scikit-learn *GridSearchCV*, with refit = True, n_jobs = 3, and verbose = 190. We then perform a stratified K-fold cross validation (Scikit-learn *StratifiedKFold*[16] with shuffle=True) of our classifier's performance across 10 splits using these optimal parameters. Finally, we use the Scikit-learn *confusion_matrix*[16] function to plot our results. Intermediate t-SNE projections were plotted using Scikit-learn *manifold.TSNE* with a perplexity = 10[16] (Supplementary Fig. 20, 22).

Raman wavenumber importance was performed using Voigt profile perturbation across all spectral wavenumbers. To achieve this, all spectra were first preprocessed as described above. Our spectra of interest (600 spectra across all 6 cellular classes) were partitioned into an 80:20 train/test split using Scikit-learn *StratifiedShuffleSplit*[16] with 10 splits. We reduce the dimensionality of our training set to 10 components using the PCA algorithm from the Scikit-learn[16]. We train our Random Forest Classifier on our training spectra using optimized hyperparameters determined using a cross-validated grid search as previously described. We iteratively perturb our test set at each wavenumber to determine the relative importance of each wavenumber to accurate spectra classification. To do this, we iterate over each wavenumber in each normalized spectrum in our test set (120 spectra per split). For each wavenumber, we perturb our test spectra with a Voigt profile curve[17, 18], varying the intensity of the Voigt function 5 times at each wavenumber for each spectrum to get a large sample set. To generate our Voigt profiles, we first take all spectra in our entire sample set (600 spectra) and shift the intensity at a given wavelength (w) to guarantee that the intensities are positive. We then randomly shuffle all intensities and randomly select one to be used to generate our Voigt profile (voigt_intensity). We generate this profile with our half-width at half-maximum (HWHM) of the Lorentzian profile, $\gamma = 2$, and the standard deviation of the Gaussian profile, $\sigma = \alpha$ / np.sqrt(2*np.log(2)), where $\alpha = 5$. From here, we create our voigt_profile = np.real(wofz((x - w + 1j*γ)/σ/np.sqrt(2))) /

σ/np.sqrt(2*np.pi), where x is the entire range of wavenumbers, and scale this distribution to range from [0,1]. We utilize the *wofz* function from the SciPy Python library to implement the Faddeeva function as the Voigt profile is related to the real part of the Faddeeva function. We also utilize various mathematical functions from the NumPy Python library to generate our profile. Voigt profile width was chosen to match peak widths seen in our spectra. To perturb our spectra with this profile, we take each spectra and shift intensities by the minimum, so that all intensities are positive (pos_spectrum). We then perturb each wavenumber by our Voigt profile to generate a modified spectrum = pos_spectrum*(1-voigt_profile) + voigt_intensity*voigt_profile. We transform this perturbed spectrum with our established PCA and classify it using our trained Random Forest Classifier. We then plot a confusion matrix for each wavenumber using Scikit-learn *confusion_matrix*[16] and generate an accuracy and f1 score using Scikit-learn *classification_report*[16], across all 6000 trials per wavenumber. Finally, we use our confusion matrix to generate the per class performance. See Supplementary Fig. 23 for more.

Supplementary Note 1: Gold nanorods for SERS applications

SERS is a phenomenon that provides Raman intensity signal enhancements of on average of 10^5-10^6, with localized hotspots providing enhancements of 10^8-10^{10} [19–21]. Commonly, SERS utilizes metallic substrates that, through their plasmonic and chemical effects (such as charge transfer ability), enhance both the electric field from the incident light and the Raman scattered light from the sample, resulting in fourth order enhancement in the local electric field $|E|^4$ [22–28]. SERS typically relies on metallic substrates to provide these enhancements. For biosensing applications, it is important for these substrates to provide large enhancements while being tunable, reproducible, stable, and inexpensive [29]. As such, colloidal nanoparticles have gained traction as one of the primary forms of metallic SERS substrates [26, 30–34]. In the realm of biosensing, gold and silver nanorods have been the primary metals used for SERS substrate synthesis due to their chemical stability and low toxicity [26, 29, 35–37]. Particularly ideal for biological sensing, nanoparticles with sharp tips, such as nanocubes, nanostars [38], nanopyramids, and nanorods, provide large Raman spectral enhancement factors, with nanorods providing the best balance of stability, reproducibility, tunability, cost, and enhancement [22, 39]. Furthermore, due to their pervasiveness, gold nanorod (GNR) synthesis and properties are well documented allowing for reproducibility and easy tunability of optical properties through choice of particle size and shape [1, 30, 40–43]. Finally, advances in SERS substrates such as nanoparticle-on-mirror (NPoM) constructs [22, 44], nanogaps and nanoholes [29, 45, 46], graphene based nanodot arrays [47], and core-shell alloys [48–50] are paving the way for future Raman-based biosensing applications.

Supplementary Note 2: Acoustic printing for handling biological samples

Acoustic printing works by using ultrasonic waves to eject a droplet from a free surface of fluid. A radio frequency (RF) burst signal is used to excite a transducer at its resonant frequency, generating ultrasonic waves that exert force on the fluid surface [51, 52]. When the focus of the transducer is aligned with the liquid-air interface and the intensity of the acoustic field is high enough, the generated radiation pressure will overcome the surface tension and the sound wave gives rise to a mound of fluid from the surface [52, 53]. If the energy of the incident wave exceeds the threshold energy, a droplet breaks free from the fluid surface at a velocity of a few meters per second due to the Rayleigh-Taylor instability [51, 54]. The droplet diameter has been shown to closely match the diffraction-limited focal width at the liquid-air interface, and as such, the droplet diameter is inversely proportional to the frequency of the transducer, with 5 MHz and 300 MHz ultrasonic waves generating droplet diameters of 300 μm and 5 μm, respectively [52]. (see Fig. 1a). ADE droplet ejection has been well characterized and has great tunability for handling a variety of biological samples [55]. Furthermore, the focused ultrasonic waves completely control the size, speed, and directionality of the ejected droplet and allow for ADE from an open liquid surface. Given that the dimensions of this open liquid surface are much larger than the diameter of the focal spot size, ADE is considered a nozzle-less technology [56]. This holds true for our downwards setup utilizing an ejection plate, given that our focal spot size is $\sim$2 orders of magnitude smaller than the 1 mm diameter hole [51, 52]. As a nozzle-less technology, ADE has unparalleled advantage in biological sample handling as compared with other commercial piezo or thermal inkjet printers that rely on physical flow focusing. In particular, ADE eliminates system clogging and compromised cell viability or biomarker structure due to shear forces generated by nozzles. Additionally, ADE relies on ultrasonic waves to generate droplets, as such, the transducer never has to contact the ejection medium, but rather can propagate through a matched coupling medium, eg. through the bottom of an acoustically “transparent” multiwell plate, with minimal loss of acoustic energy, mitigating risks of sample contamination and loss of sterility [56]. ADE has also gained traction for versatility in setup and ability for high-throughput droplet generation. For a single acoustic ejector, the limiting factor for droplet ejection rate is the dissipation of capillary waves propagating radially outwards on the fluid surface after ejection [51, 57]. Advances in ADE have led to improvements in fabrication methods of the focusing lenses and ejector arrays including: spherical lenses in silicon, spherical PZT shells, and fresnel acoustic lenses [51, 58]. These advancements have lead to the development of high-throughput ejector arrays greater than 1000 print heads and ejection rates of 25 kHz, allowing for ejection of a 10 mL of fluid in under an hour [58]. Furthermore, these advances have expanded the utility of ADE for biological samples handling to include cellular acoustic printing [55, 59, 60],

biological crystallography [61–63], high-throughput screening (HTS) of biological agents [64], and for sample preparation in MALDI [65], highlighting the vast potential for biological analysis with ADE.

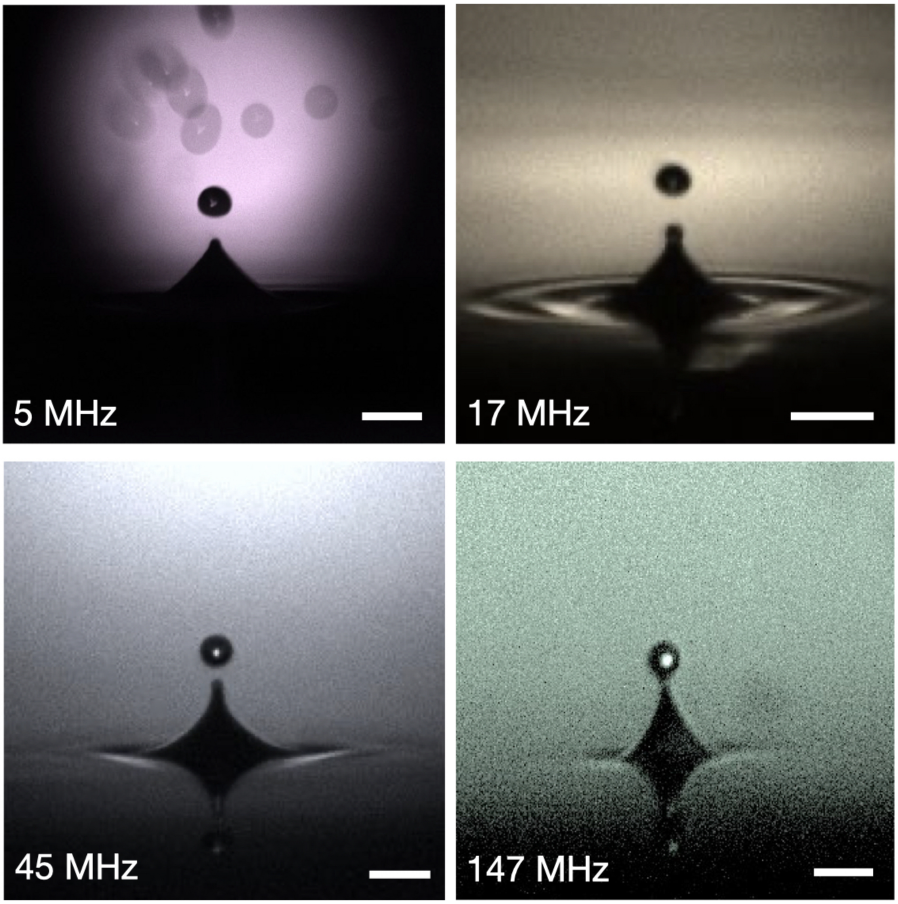

Supplementary Fig. 1 Photographs of droplets printed with a range of acoustic frequencies. Droplets were printed with 4.8 MHz, 17 MHz, 44.75 MHz, and 147 MHz and had droplet diameters of 300 μm, 84 μm, 44 μm, and 15 μm respectively, highlighting the tunability of acoustic droplet ejection. Scale bars are 500 μm, 200 μm, 100 μm, and 25 μm respectively.

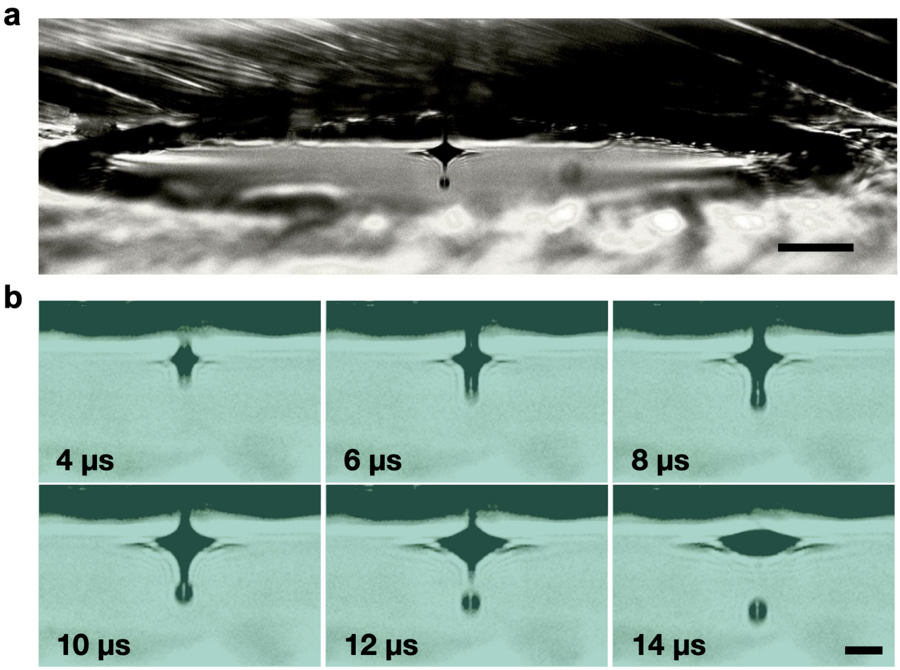

Supplementary Fig. 2 **a,** Stroboscopic photograph showing a droplet being ejected downwards through the 1 mm hole on our ejection plate. Droplet was ejected from a pool of deionized water using a transducer operating at 147 MHz, Photo was taken with a 12 μs phase delay after the burst was triggered. Scale bar is 100 μm. **b,** Stroboscopic images of the time evolution of downward droplet ejection through the 1 mm hole at an acoustic frequency of 147 MHz. Droplet shown here is 15 μm in diameter and $\sim$2 pL in volume. Droplet was ejected with 0.096 μJ of energy with a pulse width of 5.5 μs, and was ejected downwards at $\sim$3.5 m/s. Scale bar is 100 μm. All images were captured with an image exposure time of 40 ms and a droplet ejection rate of 1 kHz. As such, each frame is composed of 40 droplet ejections.

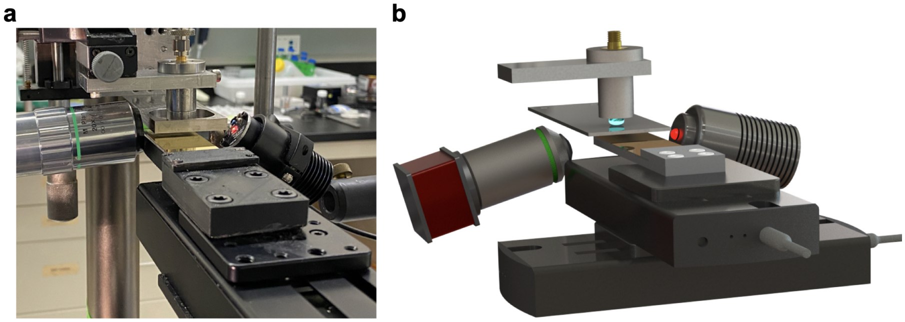

Supplementary Fig. 3 Photo **a,** and rendering **b,** showing acoustic printing setup, including camera with 20x objective, baseplate with 1 mm diameter hole, strobing LED, gold-coated glass slide mounted onto a motorized XY stage, acoustic transducer, and printing fluid (teal).

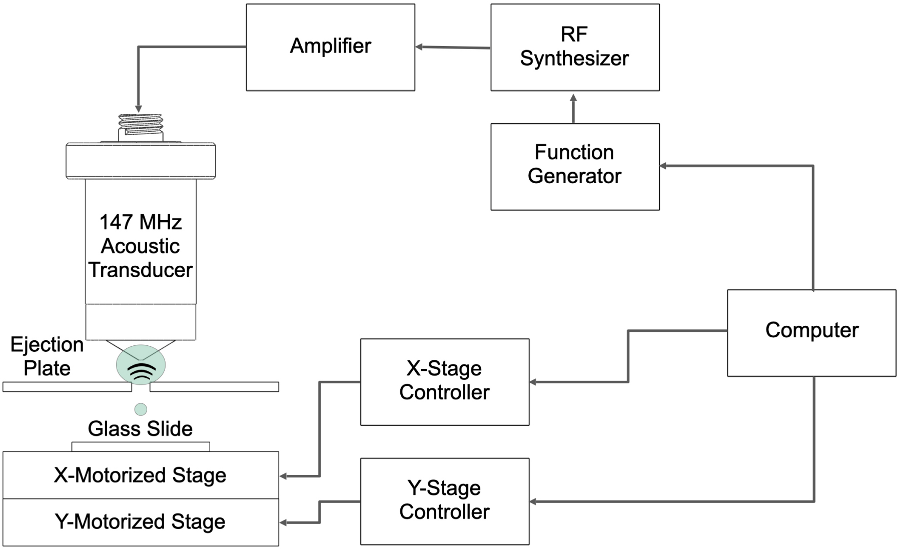

Supplementary Fig. 4 Schematic showing the acoustic droplet ejection setup. The printing fluid (teal) rests between the focused acoustic transducer and the ejection plate with the 1 mm hole, held in place through surface tension. The droplets are ejected downwards onto a gold-coated glass slide mounted onto a motorized XY stage (stacked single axis stages). The burst signals to the transducer are generated from a function generator, routed through an RF synthesizer, and finally through a power amplifier before reaching the transducer. Ejection and movement of the mounted slide are controlled synchronously using MATLAB code.

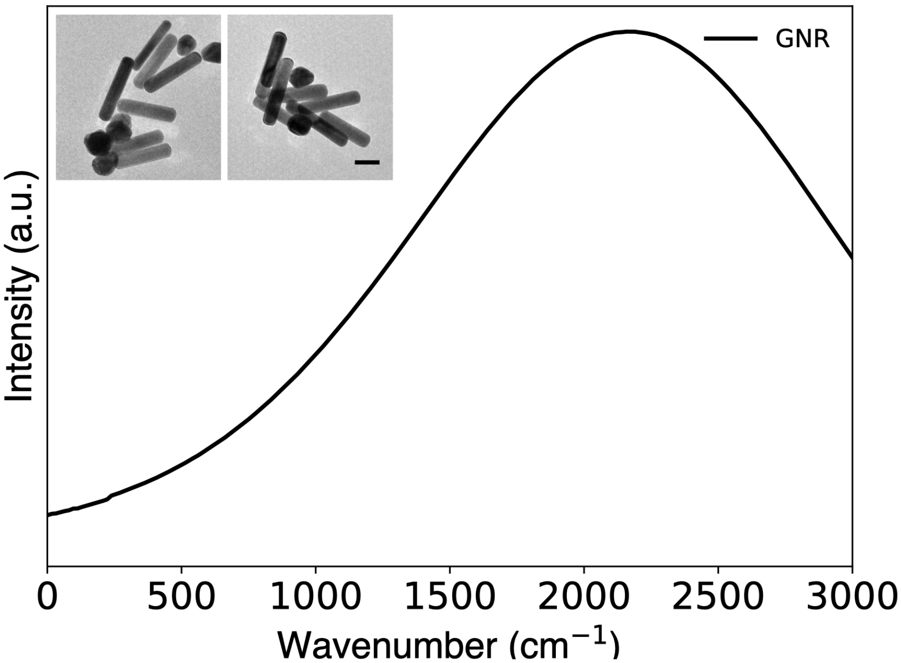

Supplementary Fig. 5 Absorption spectra of GNRs used for Raman spectroscopy. Inset shows TEM of GNRs. Scale bar is 50 nm.

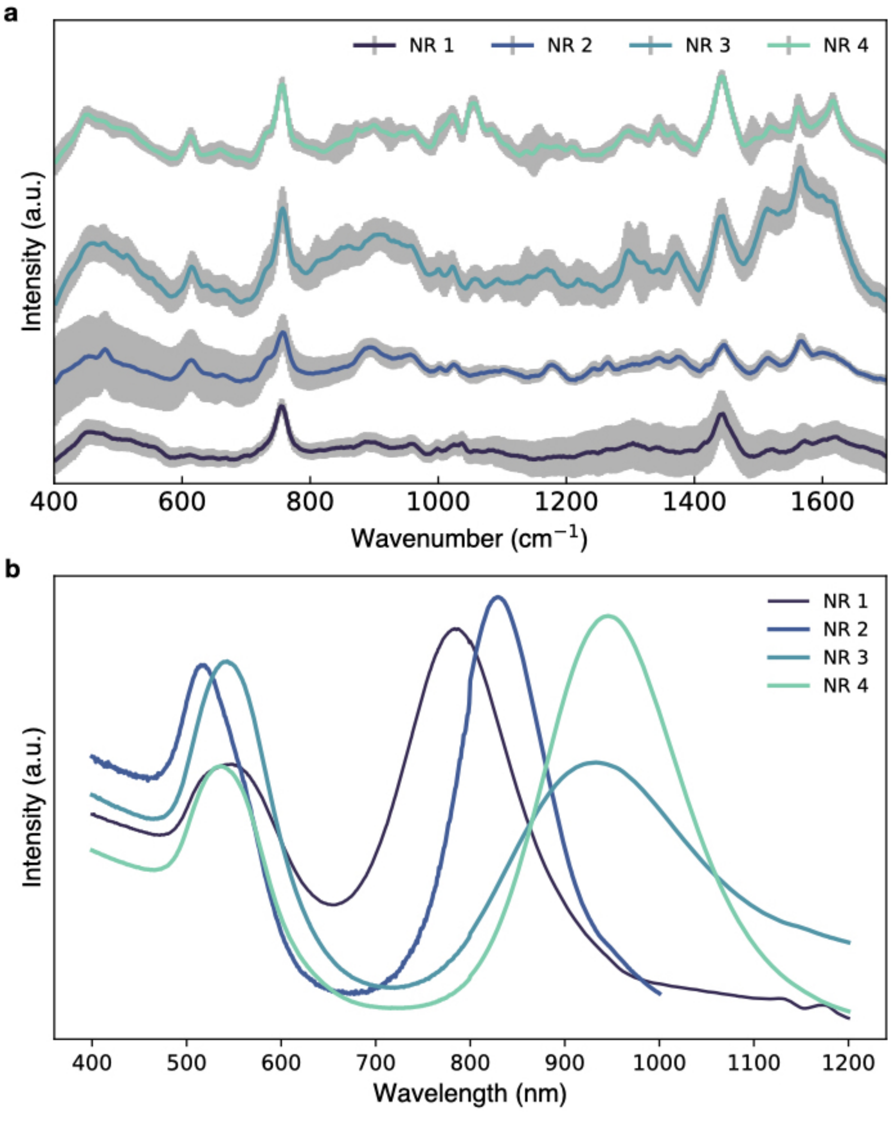

Supplementary Fig. 6 Bacterial interrogation across multiple nanorod syntheses and resonance frequencies. We synthesized 4 different batches of GNRs and evaluated bacterial droplets with each batch. **a,** UV-Vis measurements of the 4 rods showing a range of resonance frequencies between 770 nm and 960 nm. Our chosen nanorods are those listed as NR4. **b,** Average spectral intensities and standard deviations collected from droplets printed with each of our four GNR batches mixed with *S. epi* bacteria diluted to a final concentration of 1e9 cells/mL in a 1:9 mixture v/v of Invitrogen UltraPure 0.5M EDTA, Invitrogen 15575020, and Mili-Q purified water onto a silanized, gold-coated glass slide. Spectra were collected with a 10x objective lens with a 0.25 NA and ~10.6 mW power. Each droplet was interrogated with a time study of 5 time points, with each exposure lasting 15 seconds. 10 droplets were analyzed from each GNR batch for a total of 50 data points across each group. The data shows the primary *S. epi* peaks around 731 and 1317 cm^{-1}, with varying max intensities, highlighting both the robustness of our system for spectral collection as well as the potential for improvements through tuning of GNR aspect ratio.

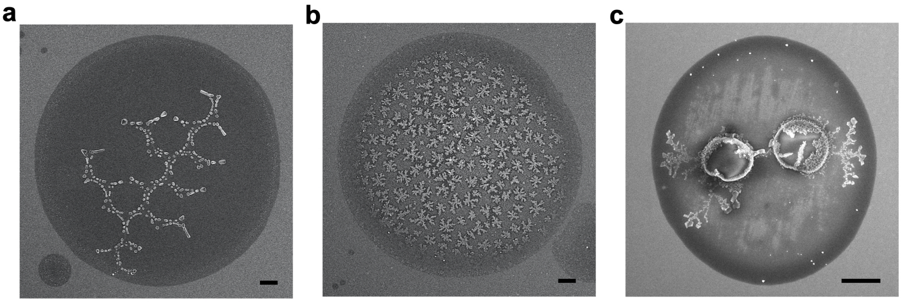

Supplementary Fig. 7 Crystallization of saline upon drying in acoustically printed droplets printed at 147 MHz. Droplets **a,** and **b,** were printed from a 10% v:v phosphate buffered saline (PBS) solution. Droplet **c,** was printed with a mixture of mouse RBCs suspended in 10% v:v PBS solution. Scale bars are 10, 10, 5 μm, respectively.

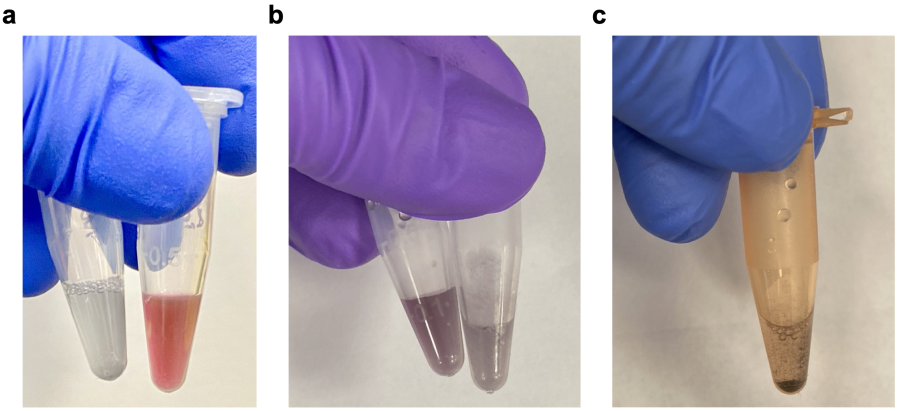

Supplementary Fig. 8 Studying the effect of the EDTA on the nanorod dispersion in fluid. **a,** Photograph showing colorimetric comparison of gold nanorods (GNR) in (left) a 1:9 mixture of Invitrogen UltraPure 0.5 M EDTA, Invitrogen 155750, and Milli-Q purified water and (right) Milli-Q purified water only. **b,** Photograph showing colorimetric comparison of GNRs mixed with *S. epi* bacteria at a concentration of 1e9 cells/mL. The image shows the gold nanorods and bacteria in (left) Milli-Q purified water only and (right) in a 1:9 ratio v/v of EDTA solution and Milli-Q purified water. **c,** Photograph showing the settling of solution of GNRs, *S. epi* at a concentration of 1e9 cells/mL, and a 1:9 ratio v/v of EDTA solution to Milli-Q purified water. Photograph was taken 5 min after the solution was mixed together. We note that these images highlight that, as expected, the EDTA appears to aggregate the GNRs into clusters. As the *S. epi* bacteria seem to cause clustering regardless of the presence of the EDTA due to their surface charge, the difference between the sample with and without the EDTA is less noticeable than in the samples of only GNRs.

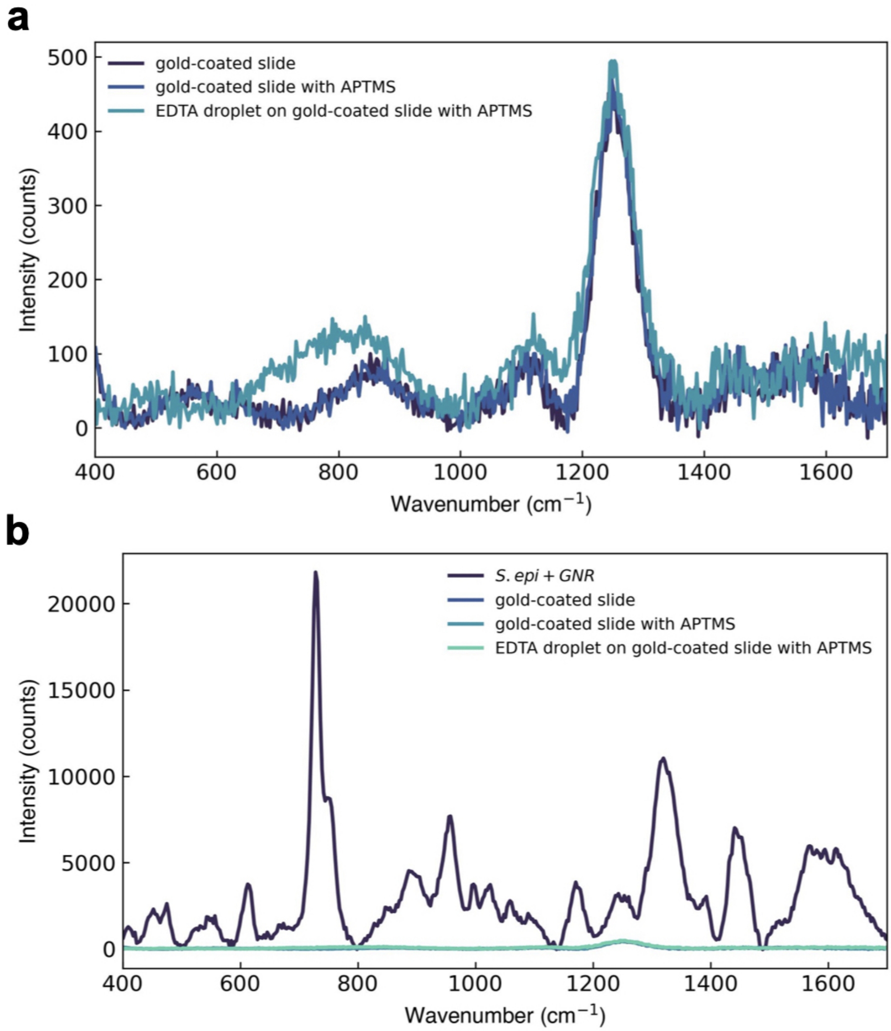

Supplementary Fig. 9 Raman of background signals. **a,** Spectra were collected of a gold-coated glass slide, a gold-coated glass slide with an APTMS silane layer, and of a droplet printed onto a gold-coated glass slide with APTMS. Droplets were printed from Invitrogen UltraPure 0.5M EDTA, Invitrogen 15575020, mixed in a 1:9 ratio v/v with Mili-Q purified water. The spectra show that most of the background signal comes from the gold substrate with little additional background from our APTMS deposition and the additional EDTA used in our cell solutions. **b,** Identical spectra to that shown in **a** overlaid with a spectrum taken of *S. epi* bacteria and GNRs suspended in EDTA solution at a concentration of 1e9 cells/mL. The plot highlights that the spectral signal intensity from our bacteria is much higher than that of the background. All spectra were collected with a 10x objective lens with a 0.25 NA and ~10.6 mW power for 15 s.

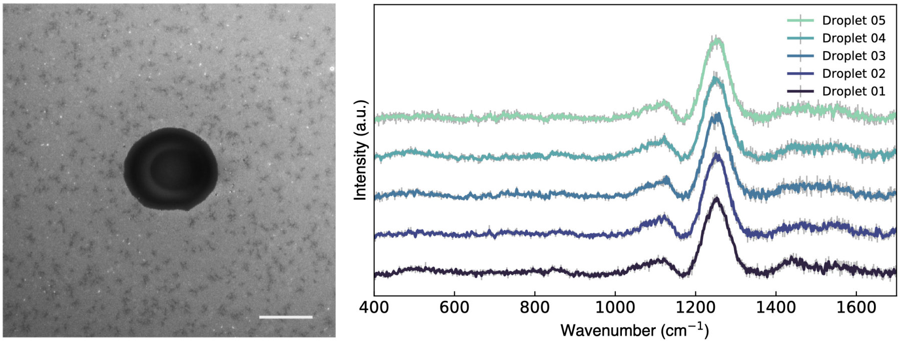

Supplementary Fig. 10 Droplets were printed from cellular dilution mixture without any cells. Droplets were printed from Invitrogen UltraPure 0.5 M EDTA, Invitrogen 15575020, mixed in a 1:9 ratio v/v with Mili-Q purified water onto a silanized, gold-coated glass slide. Spectra were collected with a 10x objective lens with a 0.25 NA and ∼10.6 mW power for 15 s. The SEM clearly shows minimal spread of the EDTA solution onto the gold-coated slide. The spectra show minimal, and consistent background signals from the EDTA solution. Scale bar is 5 μm.

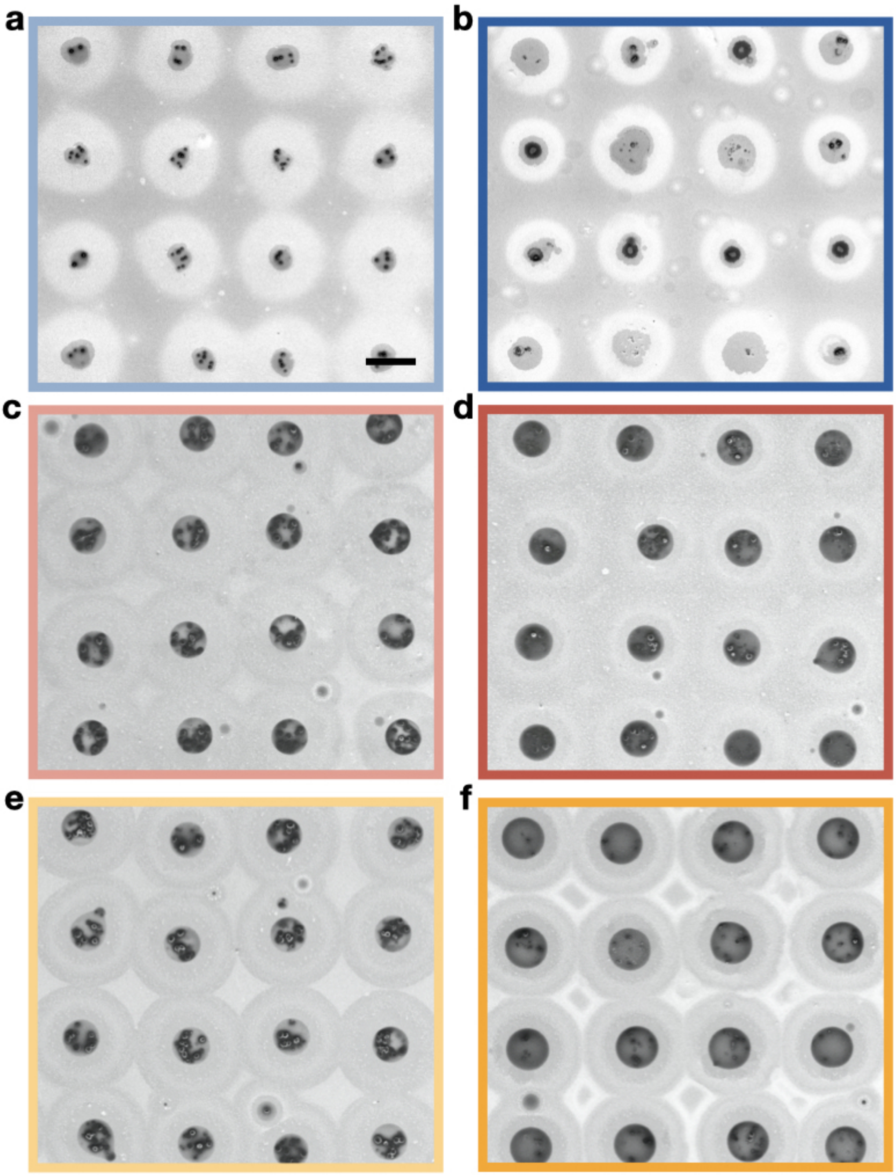

Supplementary Fig. 11 SEMs of grids printed with cell and GNR mixtures. SEMs show 16 droplets imaged out of a grid of over 400 droplets. Mixtures were printed from cells with and without GNRs diluted in a 1:9 mixture of EDTA solution and Milli-Q water to a final concentration of 1e9 cells/mL. All grids were acoustically printed using a transducer operating at 147 MHz with a 5.5 μs pulse width burst signal. SEMs show **a,** *S. epi* **b,** *S. epi* mixed with GNRs both ejected with 0.096 μJ of acoustic energy and **c,** mouse RBCs **d,** mouse RBCs mixed with GNRs **e,** 1:1 mixture of *S. epi* and mouse RBCs **f,** 1:1 mixture of *S. epi* and mouse RBCs with GNRs all printed with 0.139 μJ of acoustic energy. The lighter and darker circles in each photo highlight the outer edge of the droplet as well as the smaller volume formed from the dried EDTA mixture containing our cells and GNRs in the center of the droplet. Scale bar is 50 μm.

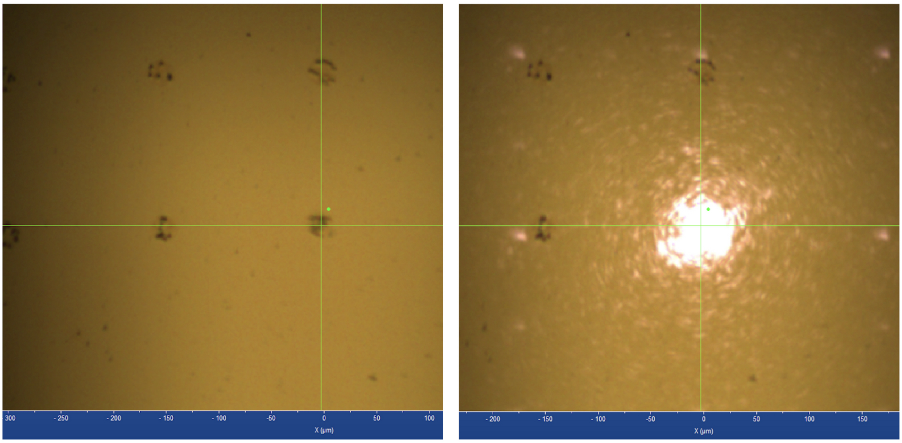

Supplementary Fig. 12 Raman focal spot size. Images show screen shots from Horiba XploRA Raman confocal microscope UI. The image on the left shows an array of droplets printed onto an APTMS silanized, gold-coated slide. Droplets were printed from a solution of gold nanorods and *S.epi* bacteria at a concentration of 1e9 cells/mL, suspended in a solution of Invitrogen UltraPure 0.5 M EDTA, Invitrogen 15575020, mixed in a 1:9 ratio v/v with Milli-Q water. The image on the right shows the same array of droplets, with the 10x objective with the 785 nm laser turned on, operating at 25% laser power or ∼10.6 mW of power. This laser spot size is ∼2 μm.

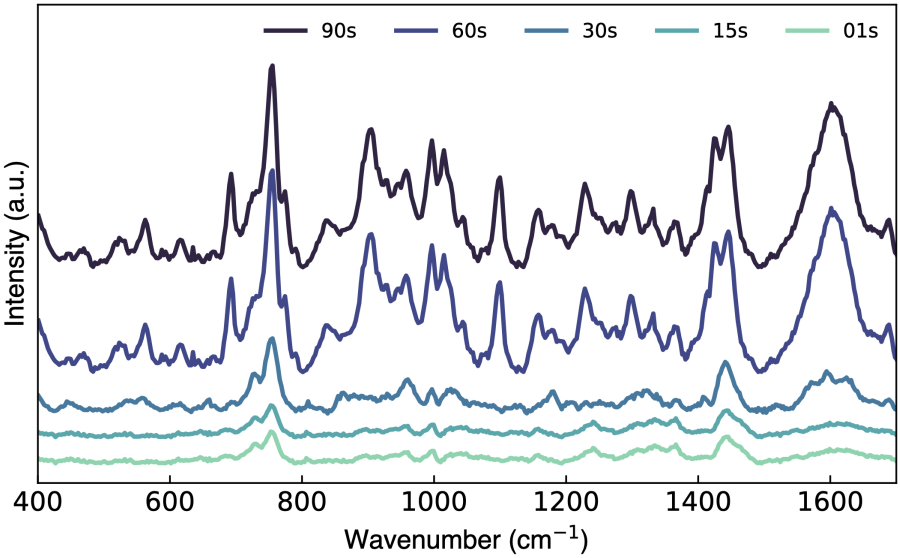

Supplementary Fig. 13 Intensity study of single droplet with *S. epi* bacteria and GNRs taken at 1, 15, 30, 60, and 90 seconds. Droplets were printed from a mixture of GNRs and *S. epi* bacteria at a final concentration of 1e9 cells/mL diluted in a 1:9 v/v of Invitrogen UltraPure 0.5 M EDTA, Invitrogen 15575020, and Mili-Q purified water onto a silanized, gold-coated glass slide. Spectra were collected with a 10x objective lens with a 0.25 NA and ∼10.6 mW power. The spectra show increasing signal intensity and signal complexity with each longer exposure time. This highlights that our time selection of 15 s is well below the time at which our sample gets damaged by the laser power. This analysis guided our choice of a 15 s acquisition time to balance gathering clear, distinct spectra with choosing a fast enough acquisition time to show potential for clinical translation.

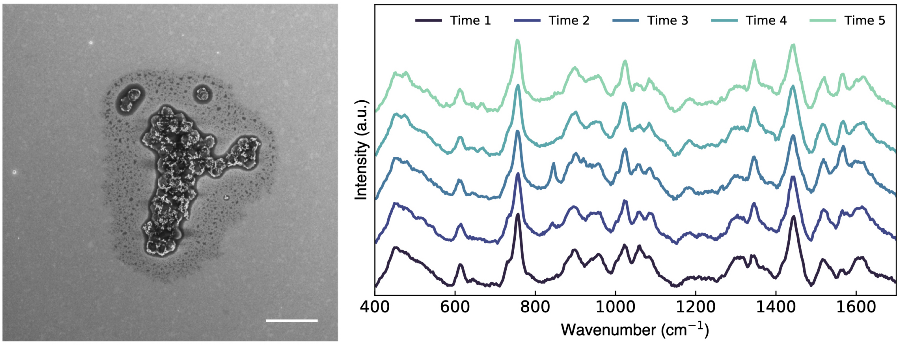

Supplementary Fig. 14 Time study of single droplet with *S. epi* bacteria and GNRs across multiple time points. Droplets were printed from a mixture of GNRs and *S. epi* bacteria at a final concentration of 1e9 cells/mL diluted in a 1:9 v/v of Invitrogen UltraPure 0.5 M EDTA, Invitrogen 15575020, and Mili-Q purified water onto a silanized, gold-coated glass slide. Spectra were collected with a 10x objective lens with a 0.25 NA and ∼10.6 mW power. Each spectrum was collected for 15 s in a time series lasting a total of 75 seconds. The SEM clearly shows a cluster of bacteria coated in GNRs. The spectra show minimal variation over the 75 second duration, showing that our acquisition time of 15 seconds does not damage the cells. Scale bar is 5 μm.

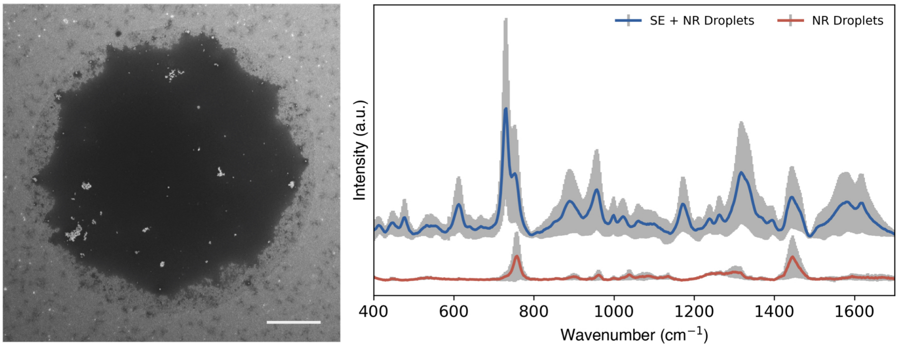

Supplementary Fig. 15 Background signal from gold nanorods (GNR). Droplets were printed from a sample containing GNRs suspended without any cells in a 1:9 v/v mixture of Invitrogen UltraPure 0.5 M EDTA, Invitrogen 15575020, and Mili-Q purified water onto a silanized, gold-coated glass slide. Spectra were collected of each droplet with a 10x objective lens with a 0.25 NA and ∼10.6 mW power for 15 s. The SEM shows a droplet containing a few clusters of GNRs clearly distinguishable, highlighting both the presence of the GNRs and the absence of a coffee-ring of nanorods. The plot shows the mean and standard deviation of 100 droplets printed from a sample of *S. epi* bacteria with GNRs in EDTA solution (in blue, identical to that from Fig. 3), and the mean and standard deviation of 20 droplets printed from the GNR solution without cells. The spectra show that while the GNRs have a background signal, hypothesized to be from any remaining CTAB present in the solution after rinsing the rods, the cells have a clearly distinguishable signal separate from that of the GNRs, similar to our baseline *S. epi* spectra. Scale bar is 5 μm.

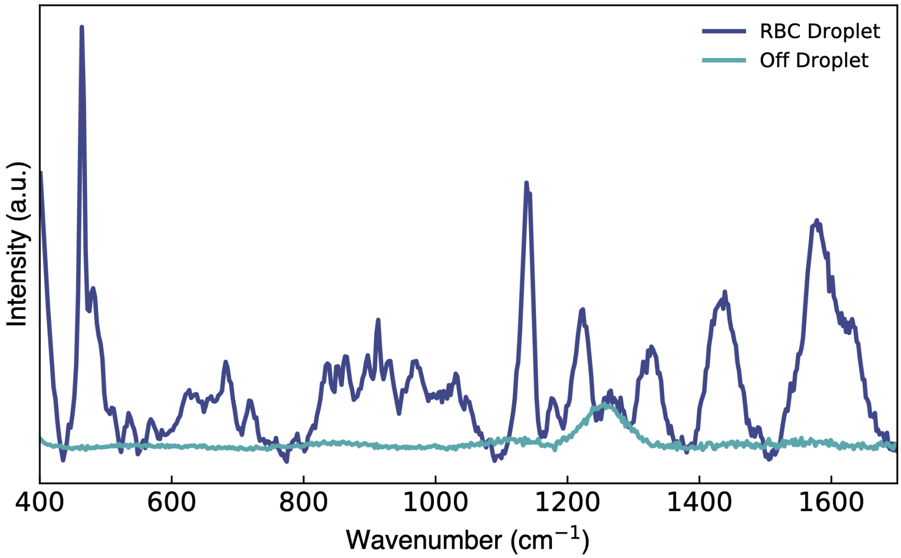

Supplementary Fig. 16 Spectra were collected from a sample of droplets printed from GNRs mixed with mouse RBCs at a final concentration of 1e9 cells/mL diluted in a 1:9 ratio v/v of Invitrogen UltraPure 0.5 M EDTA, Invitrogen 15575020, and Mili-Q purified water onto a silanized, gold-coated glass slide. Spectra were collected with a 10x objective lens with a 0.25 NA and ~10.6 mW power for 15 s. The first spectra is taken while the focal spot is centered on the droplet while the other is taken when on the silanized gold substrate to the side of the droplet highlighting that our signal is coming directly from the droplet and not from any background material on the slide.

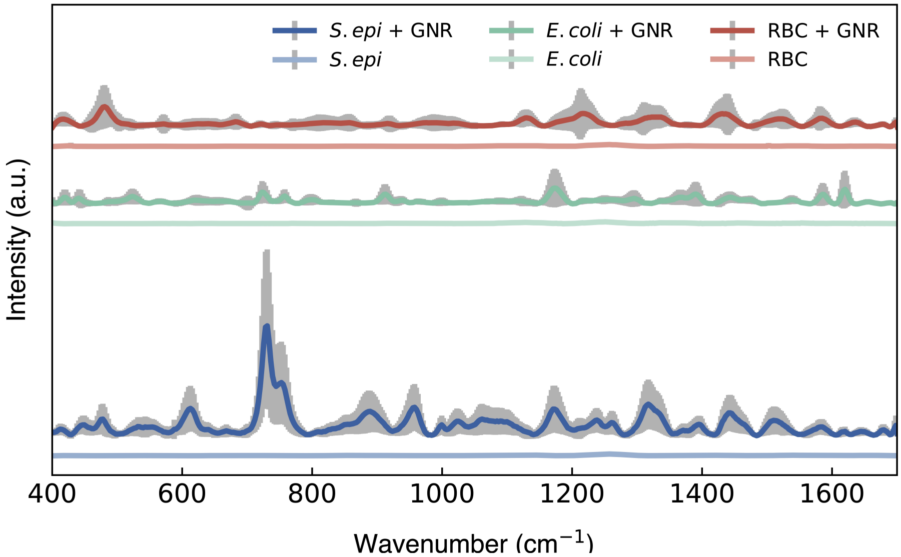

Supplementary Fig. 17 Plot showing the mean and standard deviation of SERS spectra taken from droplets printed from three cell lines (*S. epi*, *E. coli*, and RBCs) with and without GNRs. Spectra were collected from 100 and 15 droplets, respectively. The plots highlight both the enhancements generated with the presence of GNRs as well as the variations in peak spectra intensity due to the variations in surface charge density on each cell line and subsequently the cells' varying attraction to the positive surface charge of the GNRs, resulting from the CTAB surfactant on their surface [2].

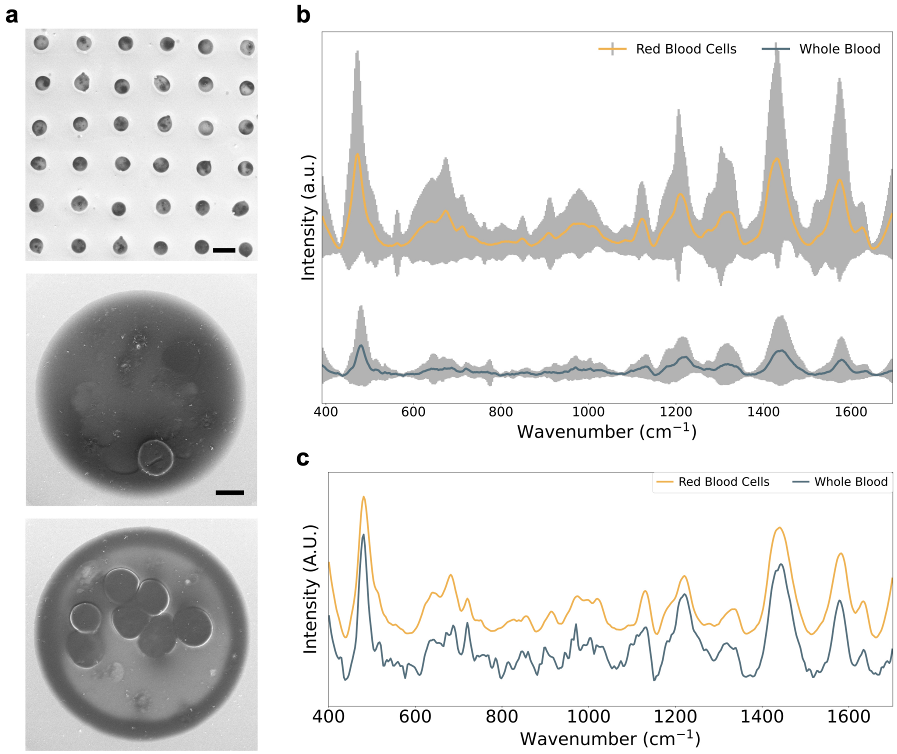

Supplementary Fig. 18 Data from droplets print from mouse whole blood mixed in with GNRs. Mouse whole blood was purchased with the addition K2EDTA as an anti-coagulating agent for processing and shipment. Blood was then diluted 1:9 with our EDTA solution (1:9 ratio v/v of Invitrogen UltraPure 0.5 M EDTA, Invitrogen 15575020 in DI water) for a final RBC count of 1.1 e9 cells/mL, closely approximating the RBC count of samples printed with RBC mixtures. GNRs at a concentration matching that all other cellular samples was added to the solution and then samples were printed onto a gold-coated slide and Raman spectra were collected. **a,** SEMs showing stable printing of solutions from diluted mouse whole blood mixed with GNRs. Top image shows a 6x6 grid of droplets as a subset of the hundreds printed. The remaining two SEMs show two representative droplets from the sample with 1 and 7 RBC, respectively. Scale bars are 50 μm and 5 μm. **b,** Mean spectra standard deviation of 100 spectra collected from samples printed from mouse RBCs and mouse whole blood, both diluted in aqueous EDTA to a final RBC count of 1e9 cells/mL mixed with GNRs. Spectra are shown after baseline correction **c,** Normalized mean spectra from data shown in **b**. Normalized means show that while the whole blood exhibits similar peaks to that of the RBCs, there are many more minor peaks in the spectrum, highlighting expected sample complexity from the whole blood sample.

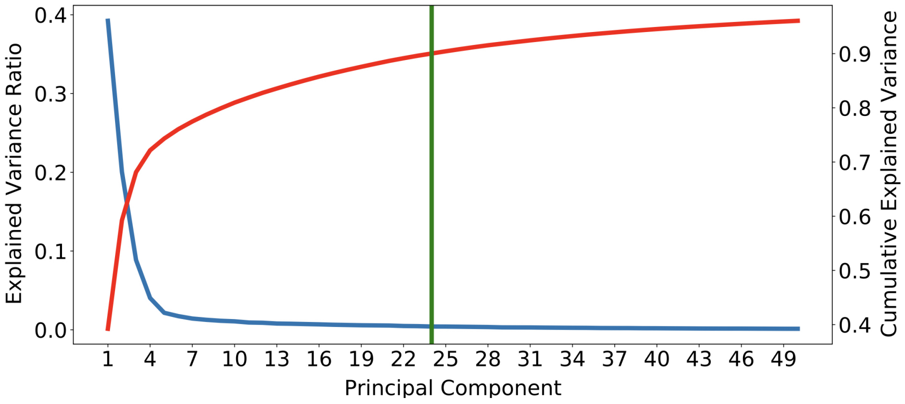

Supplementary Fig. 19 Plot of the percentage of variance attributed to each principal component and the cumulative explained variance over 50 components. The green line indicates the number of PCA components necessary to capture 90% of all explained variance in our samples. For all 300 spectra from our single cell-line droplets, we demonstrate that we can account for at least 90% of all variance with 24 components generated from all 508 wavenumber features in our spectra.

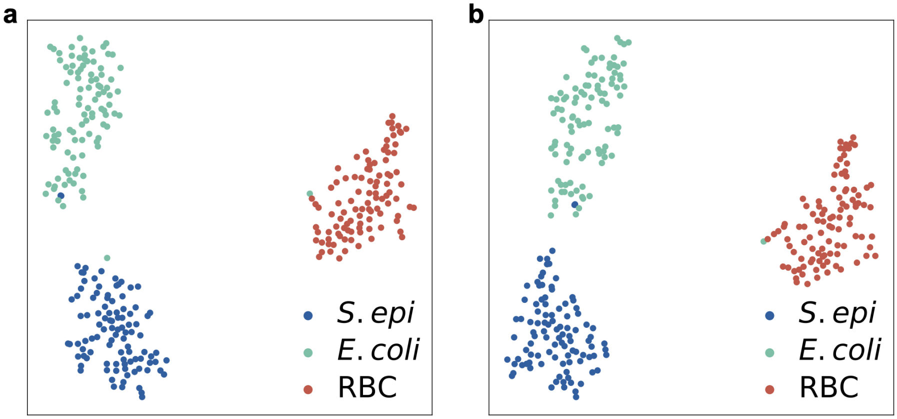

Supplementary Fig. 20 Plots showing a 2-component, t-distributed stochastic neighbor embedding projection (t-SNE) with perplexity = 10 across our 3 single cell-line classes. Data is plotted **a,** with data inclusive of all wavenumber features and **b,** after performing a 24-component PCA for dimensionality reduction. Plots show relative clustering of our classes and minimal variation to clustering after dimensionality reduction.

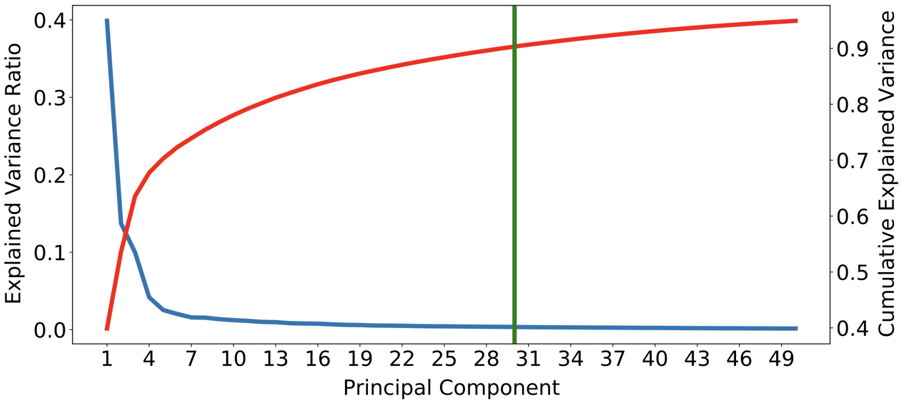

Supplementary Fig. 21 Plot of the percentage of variance attributed to each principal component and the cumulative explained variance over 50 components. The green line indicates the number of PCA components necessary to capture 90% of all explained variance in our samples. For all 600 spectra from our 3 single cell-line droplet classes and our 3 cell mixture classes, we demonstrate that we can account for at least 90% of all variance with 30 components generated from all 508 wavenumber features in our spectra.

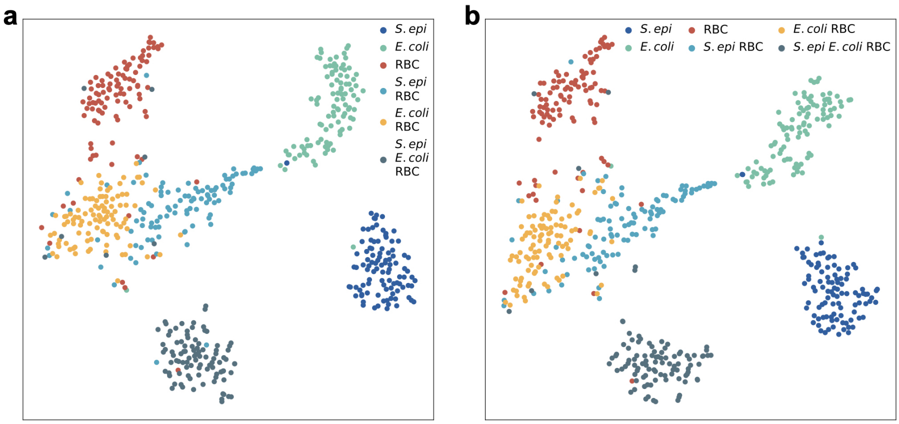

Supplementary Fig. 22 Plots showing a 2-component, t-distributed stochastic neighbor embedding projection (t-SNE) with perplexity = 10 across all 6 of our classes. Data is plotted **a,** with data inclusive of all wavenumber features and **b,** after performing a 30-component PCA for dimensionality reduction. Plots show relative clustering of our classes and minimal variation to clustering after dimensionality reduction.

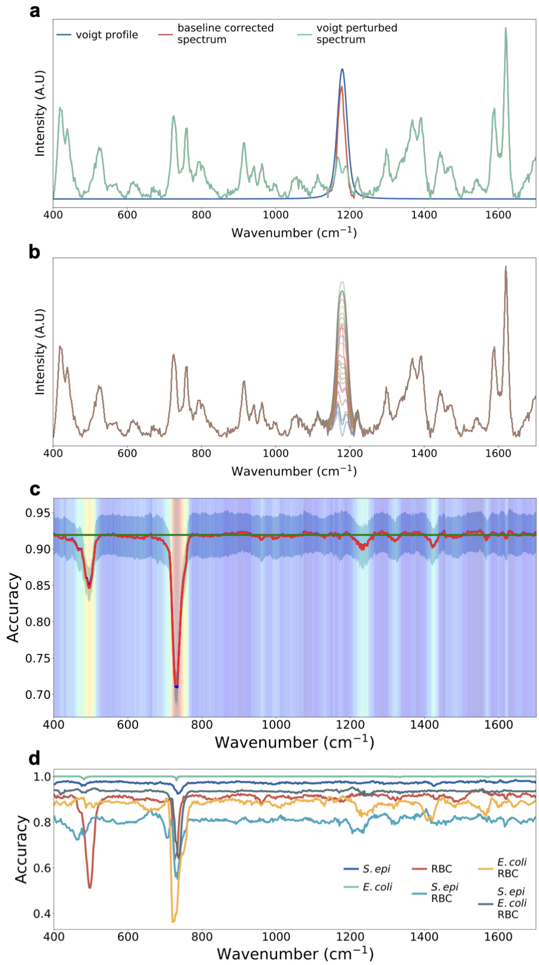

Supplementary Fig. 23 Feature Validation. We perform feature validation on our spectra to determine which wavenumbers and spectral bands are most important for our classifier. We take our 600 spectra across all 6 cellular classes and split the samples using a stratified shuffle split into an 80:20 train/test split. For each spectrum in our training set, we iteratively perturb the spectrum at each wavenumber. After each perturbation, we calculate the classification accuracy and compare with our baseline accuracy. Every wavenumber of each spectrum in the test set is perturbed 5 times and all results are averaged for our final feature extraction. Spectra were perturbed with a normalized Voigt profile. Line width chosen to roughly match peak widths seen in our spectra. **a,** plot showing an example Voight curve (blue), unperturbed example spectrum from our dataset (red), and perturbed spectrum (green). **b,** Voigt profile intensities were chosen through random sampling of all spectra in our training set. Plot shows an example of a spectrum from our sample set with 100 different perturbations. **c,** Heatmap highlighting feature validation performed to determine relative weight of spectral wavenumbers in our Random Forest classification. Heatmap is overlaid with a plot of mean and standard deviation of the perturbed classification accuracy (red) and f1 score (blue) calculated across all trials. Mean accuracy is plotted in green. Wavenumbers with lower accuracies are shown to be critical features, as random perturbations in these regions are highly correlated with decreases in classification accuracy. **d,** Plot of the mean classification accuracy broken down into accuracies across each of our cellular and mixture classes.

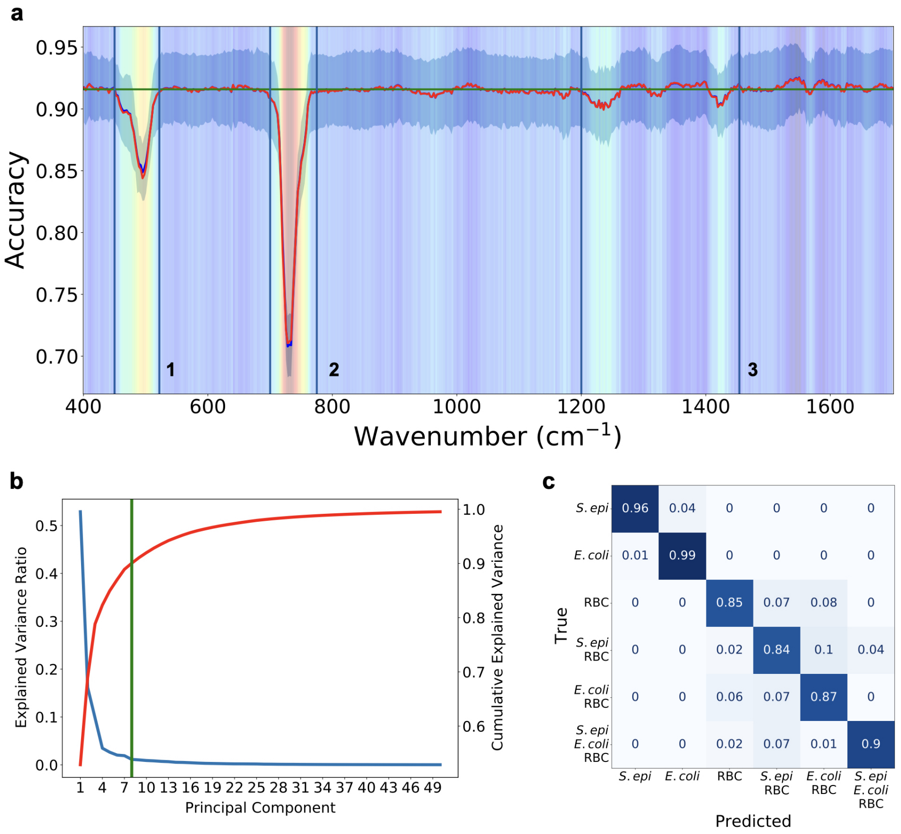

Supplementary Fig. 24 Classification using spectral feature bands of interest evaluated across 600 spectra collected from single cell-line droplets of *S. epi*, *E. coli*, and mouse RBCs mixed with GNRs, and our 3 cell mixtures. **a,** Heatmap presented in Supplementary Fig. 22, highlighting feature importance calculations performed to determine relative weight of spectral wavenumbers in our random forest classification. Heatmap is overlaid with 3 bands representing key spectral bands used by our classifier. We further demonstrate that these bands are primarily responsible for our classification accuracies by preprocessing our spectra by removing spectral features outside these bands (420-522 cm^{-1}, 700-775 cm^{-1}, 1200-1454 cm^{-1}). We then reduced the dimensionality of our remaining features using an 8-component PCA as previously reported. **b,** Plot of the percentage of variance attributed to each principal component and the cumulative explained variance over 50 components. The green line indicates the number of PCA components necessary to capture 90% of all explained variance in our samples. For this sample set taking only specific wavenumber bands from our spectra, we demonstrate that we can account for at least 90% of all variance with only 8 components generated from all 508 wavenumber features in our spectra. **c,** Finally, we use our previously described random forest classifier on our samples and perform a stratified K-fold cross validation of our classifier's performance across 10 splits. Results are plotted on a normalized confusion matrix. We show that we achieve $\geq$ 81% classification accuracy across all samples as compared with the $\geq$ 87% classification accuracy achieved when evaluating the entire spectra window from 400-1700 cm^{-1}. These results further validate our feature recognition model. Furthermore, they pave the way for future development of low cost POC systems by demonstrating that the use of low-cost spectrometers with limited spectral windows may be possible for such diagnostic work.

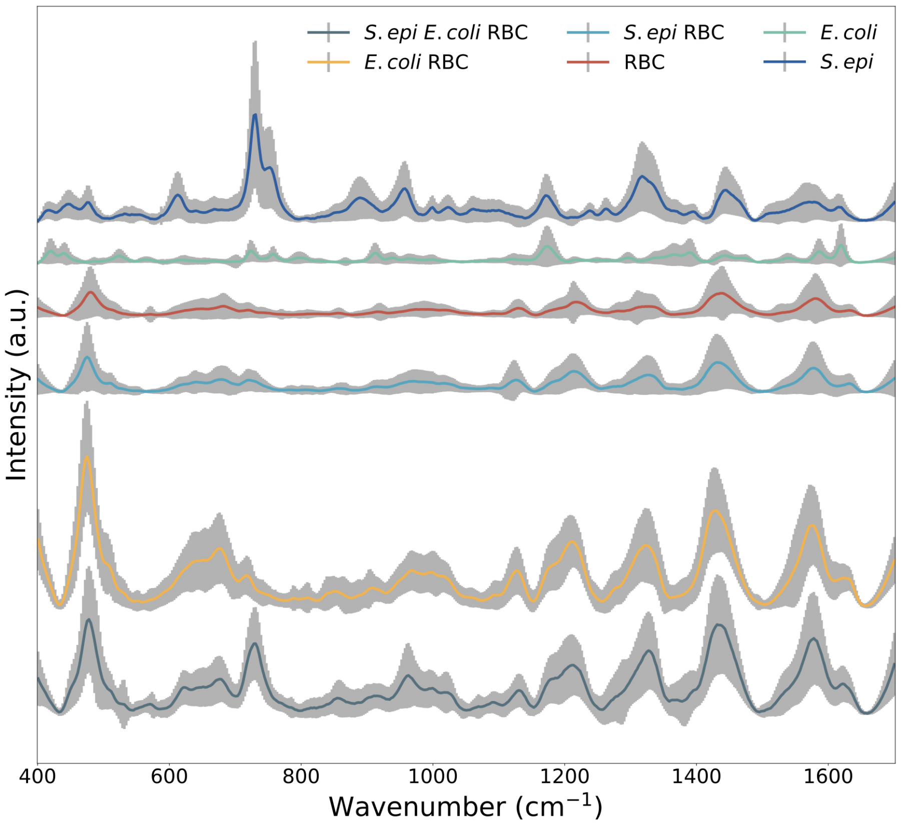

Supplementary Fig. 25 Plot showing the mean and standard deviation of SERS spectra taken from droplets printed from our 6 droplet classes: three single-cell line classes (*S. epi*, *E. coli*, and RBCs) and three mixture classes (equal mixtures of *S. epi* and RBCs, *E. coli* and RBCs, and *S. Epi*, *E. coli*, and RBCs) all diluted to a final concentration of 1e9 cells/mL of each cell type in our aqueous EDTA solution and mixed with GNRs. Spectra were collected from 100 droplets for each class.

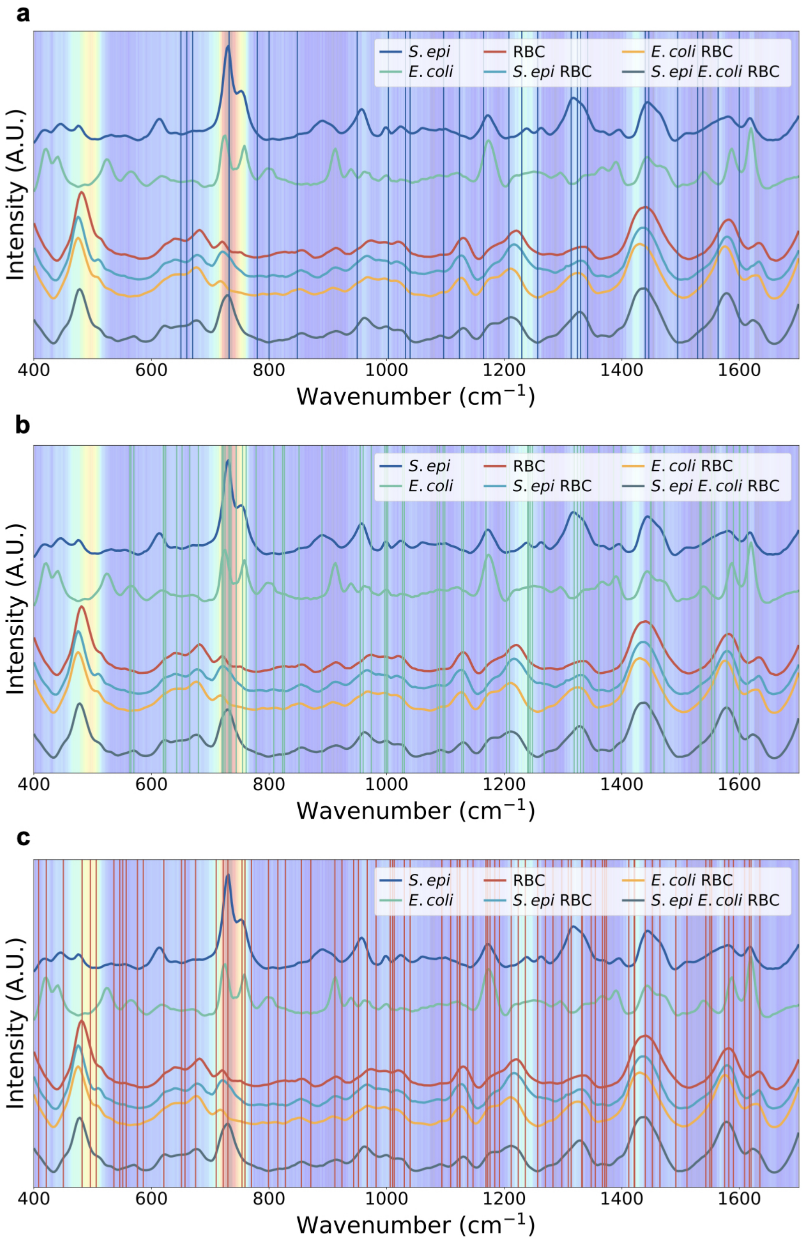

Supplementary Fig. 26 Heatmap highlighting feature extraction performed to determine relative weight of spectral wavenumbers in our random forest classification. Heatmap is overlaid with the mean SERS spectra of 100 measurements each, taken from single droplets printed from three cell lines (*S. epi*, *E. coli*, and RBCs) and three mixtures (*S. epi* and RBCs, *E. coli* and RBCs, and *S. epi*, *E. coli*, and RBCs) mixed with GNRs. Wavenumbers representative of biological peaks of dried and liquid SERS of **a,** *S. epi*, **b,** *E. coli*, and **c,** RBCs previously reported in the literature are plotted as vertical lines [2, 66–76].

Supplementary Table 1: Tentative band assignments of the SERS spectra of *S. epi*, *E. coli*, RBCs as reported in literature.

Peak Pos. (cm^{-1})	Cell Type	Peak Assignment	Peak Pos. (cm^{-1})	Cell Type	Peak Assignment
408	RBC	$\delta(C_\beta C_a C_b)4 + \delta(C_\beta Me)$[74]	1124	*S. epi*	$\nu(PO_2)$[76]
421	RBC	δ(Fe–O–O)[73]		RBC	ν13 or ν42[74]
450	RBC	Fe–O[73]	1125	RBC	Proteins: C–N stretch and C–C stretch[73]
482	RBC	γ12[73]	1128	*E. coli*	Amide III, adenine, polyadenine, and DNA[72]
496	RBC	γ12[74]	1138	RBC	ν(C_β-Methyl),ν5[73]
506	RBC	Fe–O, Proteins: S–S stretch[73]	1147	RBC	Proteins, lipids: C–N and C–C stretch[73]
536	RBC	γ21, ν25, Proteins: S–S stretch, skeletal deform[73]	1165	*S. epi*	Tyr, Phe, amide III[76]
546	RBC	ν(Fe–O–O)[73]	1169	RBC	ν30[74]
551	RBC	ν49 or ν(Fe–O-O)[74]		*E. coli*	12-methyltetradaconic acid or 15-methylpalmitic acid or acetoacetate[66]
557	RBC	$\nu(Fe - O - O)$[73]	1172	RBC	ν(pyr half-ring)$_{asym}$, ν30[73]
565	*E. coli*	C-S-S-C[68]	1176	RBC	C-H bending, Tyr[75]
563	*E. coli*	Carbohydrates[72]	1184	RBC	Thr: CH_3 rocking[73]
570	*E. coli*	Carbohydrates[69]	1192	RBC	Thr: CH_3 rocking[73]
576	RBC	ν(Fe–O_2)[73]	1204	*E. coli*	Phe[76]
586	RBC	ν48[74]	1208	*S. epi*	Phe[76]
620	*E. coli*	Phe[68]	1209	*E. coli*	Aromatic amino acids in proteins[69]
621	*E. coli*	C-C twisting mode of Phe[76]	1212	RBC	δ(C_mH), ν13 or ν42[73]

Peak Pos. (cm^{-1})	Cell Type	Peak Assignment	Peak Pos. (cm^{-1})	Cell Type	Peak Assignment
	RBC	ν12[74]	1224	RBC	$\delta(C_mH)$[73]
					ν13 or ν42[73, 74]
624	*E. coli*	Aromatic ring skeleton[72]	1230	*S. epi*	$\nu(PO_{2-})$, amide III[76]
643	*E. coli*	Guanine ring breathing[76]	1235	*E. coli*	Vibration of N-H[67]
650	*S. epi*	Guanine ring breathing[76]	1236	RBC	Trp: ring[73]
651	RBC	Cys: C–S stretch[73]	1240	*E. coli*	Amide III[68]
652	*E. coli*	δ(COO-)[72]	1241	*E. coli*	$\nu(PO_{2-})$, amide III
657	RBC	$\delta(\text{pyr deform})_{sym}$, ν7[73]	1248	*E. coli*	CH_2 stretching[66]
659	*E. coli*	Guanine (C-S)[66]	1257	*S. epi*	Amide III[76]
660	*S. epi*	Guanine, thymine ring breathing[76]		RBC	Glu: CH_2 wag; proteins, lipids: amide III, $\delta(CH_2/CH_3)$[73]
665	*E. coli*	NAG (N-acetylglucosamine)[76]	1268	*E. coli*	$\delta(CH_2)$ Amide III[72]
670	*S. epi*	NAG (N-acetylglucosamine)[76]	1270	RBCs	Proteins, lipids: amide III, $\delta(CH_2/CH_3)$[73]
675	RBC	ν7[74]	1283	RBC	$\delta(C_mH)$, ν21[73]
680	*E. coli*	Adenine[69]	1298	RBC	$\delta(C_mH)$, ν21[73]
710	RBC	ν11[74]	1309	RBC	ν21[74]
720	*E. coli*	Adenine in Flavin adenine dinucleotide (FAD) and Nicotinamide Adenine Dinucleotide (NAD)[68]	1314	*S. epi*	Guanine, CH_2 twist (lipids)[76]
722	*E. coli*	Adenine[66]		RBC	Phe, Glu, Ser, Met, His: CH_2 wag[73]
	RBC	Amino acids: δ(COO-)[73]	1319	*E. coli*	Guanine, CH_2 twist (lipids)[76]
725	*E. coli*	Adenine ring breathing[76]	1324.5	*S. epi*	Protein and carboxylic stretches[71]

Peak Pos. (cm^{-1})	Cell Type	Peak Assignment	Peak Pos. (cm^{-1})	Cell Type	Peak Assignment
730	RBC	Amino acids: δ(COO-)[73]		*E. coli*	Protein and carboxylic stretches[71]
731	*S. epi*	Adenine part of the flavin derivatives or glycosidic ring mode of polysaccharide[70]	1330	*S. epi*	Adenine part of the flavin derivatives or glycosidic ring mode of polysaccharides[70]
	E. coli	Adenine part of the flavin derivatives or glycosidic ring mode of polysaccharides[70]		*E. coli*	Adenine part of the flavin derivatives or glycosidic ring mode of polysaccharides[70]
					$\nu(NH_2)$ adenine, polyadenine, DNA[72]
					CH_2/CH_3 wagging mode in purine bases of nucleic acids[68]
732.5	*S. epi*	purine ring-breathing modes[71]	1332	RBC	ν41[74]
735	*E. coli*	adenine, glycosidic ring mode[72]	1333	RBC	ν(pyr half-ring)$_{sym}$, Proteins: δ(CH); CH_2, CH_3 wag[73]
744	*E. coli*	B_{1g} heme vibration (cytochrome c)[76]	1335	*E. coli*	CH_2 deformation or Trp[66]
					Protein twisting (CH_2 and CH_3), $\nu(NH_2)$ adenine[67, 76]
754	RBC	ν15[74]	1342	*S. epi*	Protein twisting (CH_2 and CH_3), $\nu(NH_2)$ adenine[76]
		Porphyrin ring breathing[75]			
755	*E. coli*	Trp ring breathing[76]	1348	RBC	Glu, Asp, Asn, Gln: CH_2 sciss, Ala, Leu, Val, Ile: CH_3 deform[73]
759	RBC	ν(pyr breathe), ν15[73]	1355	*E. coli*	Ch deformation vibrations[67]

Peak Pos. (cm^{-1})	Cell Type	Peak Assignment	Peak Pos. (cm^{-1})	Cell Type	Peak Assignment
761	*E. coli*	Ring I deformation[69]		RBC	Glu, Asp, Asn, Gln: CH_2 sciss, Ala, Leu, Val, Ile: CH_3 deform[73]
770	RBC	Trp: indole sym. breathe[73]	1361	*E. coli*	=CH in plane (lipid) or amide III (protein)[69]
778	*E. coli*	DNA/RNA ring breathing (cytosine/thymine)[76]	1367	RBC	Half ring stretching, porphyrinReokrungruang2019-qj
780	*S. epi*	DNA/RNA ring breathing (cytosine/thymine)[76]	1368	*E. coli*	ν(COO-) and δ(C-H) proteins[76]
799	RBC	ν(pyr breathe), $\nu 6$[73]	1371	RBC	$\nu 4$[74]
800	*S. epi*	DNA/RNA ring breathing	1374	RBC	ν(pyr half-ring)$_{sym}$, $\nu 4$, Proteins, lipids: $\delta(CH_3)_{sym}$[73]
808	*E. coli*	ν(CN) Tyr, Val[72]	1386	*E. coli*	$\delta(CH_3)$ symmetrical[76]
815	RBC	Ser: γ(COO-)[73]	1399	*E. coli*	C-O-O- stretching in amino acids[76]
823	*E. coli*	Different C-N stretch[69]	1412	RBC	ν(pyr quarter-ring)$_{sym}$, $\nu 20$, Proteins: COO- sym stretch[73]
826	*E. coli*	V_a(O-P-O) str.[76]	1421	RBC	$\nu(C\_alpha C_m)_{sym}$
828	RBC	γ(CmH)[73]	1422	RBC	$\nu 28$[74]
848	*S. epi*	Thymine[76]	1440	*S. epi*	protein or lipid[71]
851	*E. coli*	Thymine[76]		RBC	CH_2 deformation - lipid, protein[75]
855	RBC	γ-Porphyrin[73]	1446	*S. epi*	Scissoring (fatty acids, phospholipids, and mono- and oligo-saccharides); CH_2/CH_3 deformation[76]

Peak Pos. (cm^{-1})	Cell Type	Peak Assignment	Peak Pos. (cm^{-1})	Cell Type	Peak Assignment
871	RBC	$\gamma(C_mH)$[73], $\gamma10$	1449	*E. coli*	Scissoring (fatty acids, phospholipids, and mono- and oligo-saccharides); CH_2/CH_3 deformation[76]
912	RBC	Glu, Ile, Thr, Lys: C–C stretch[73]	1450	*E. coli*	CH_2/CH_3 deformation of proteins and lipids[68]
924	RBC	Amino acids: C-COO- stretch[73]	1452	RBC	$\delta(CH_2/CH_3)$, Lipids:$\delta(CH_2/CH_3)$[73]
944	RBC	$\nu46$[74]	1465	RBC	$\nu(C_\alpha C_m)_{sym}$, $\nu3$, Lipids:$\delta(CH_2/CH_3)$[73]
950	*S. epi*	$\nu(CH_3)$ of proteins (a-helix)[76]	1472	*E. coli*	CH_2 deformation of the protein molecules[66, 67]
952	RBC	$\gamma(C_aH=)$[73]	1492	RBC	trp:indole ring bend, indole CH bend[73]
955	*E. coli*	$\nu(CH_3)$ of proteins (a-helix)[76] $\nu(CN)$[72] C=C deformation[68]	1495	*S. epi*	$\delta(CH_2)$[76]
960	*E. coli*[66]	δ(C=C) or tyrosine	1511	RBC	$\nu38$[74]
967	RBC	Proteins: C–C stretch[73]	1529	*S. epi*	Amide II of proteins, N-acetyl related bands (amide II)[76]
974	*E. coli*	C=C deformation[69]	1533	*E. coli*	Amide II of proteins, N-acetyl related bands (amide II)[76]
982	RBC	$\gamma(C_aH=)$[73]	1535	*E. coli*	adenine, cytosine, and guanine[76]
997	*E. coli*	Phe or glucose[66]	1538	*S. epi*	Amide II of proteins[76]

Peak Pos. (cm^{-1})	Cell Type	Peak Assignment	Peak Pos. (cm^{-1})	Cell Type	Peak Assignment
1000	*E. coli*	Phe[76]	1543	RBC	$\nu 11$[74]
1002	*S. epi*	Phe[76]	1550	RBC	$\nu(C_\beta C_\beta)$, $\nu 11$, Proteins, lipids: amide II, Trp[73]
1006	RBC	Phe: indole asymmetric ring breathe[73]	1553	*E. coli*	Amide II of proteins[76]
1010	RBC	$\nu 45$[74]		RBC	N-H, Trp[75]
1013	RBC	Trp: indole asymmetric ring breathe[73]	1558	*E. coli*	CH_2 deformation[69]
1027	*E. coli*	A Ring stretching or (C-H) deformation[66]	1564	*S. epi*	Amide II of proteins, guanine/adenine[76]
1030	*E. coli*	Phe: C-H in plane bending[76]	1575	RBC	skeletal mode, trp[73]
	RBC	Phe: in-plane ring CH deform[73]	1579	*E. coli*	Guanine, adenine, trp (proteins)[75]
1032	*S. epi*	Phe: C-H in plane bending[76]	1582	RBC	C-C asymmetric stretching, hemoglobin[75]
1040	*S. epi*	ν(CC) aromatic ring[76]	1590	*E. coli*	Phe, hydroxyproline, Tyr[68]
	RBC	$\delta(=C_bH_2)_{asym}$[73]		RBC	$\nu(C_\alpha C_m)_{asym}$, $\nu 37$, Phe, Tyr[73]
1056	*E. coli*	Stretching vibration of C-C in alkanes[67]	1600	*S. epi*	Protein and carboxylic stretches[71]
1086	*E. coli*	Phe[66]		*E. coli*	Protein and carboxylic stretches[71]
1090	*E. coli*	C-C skeletal and C-O-C stretching from glycosidic link[68]	1601	*E. coli*	Tyr, C-N stretching vibration
1094	RBC	$=C_{2vinyl}H$[73]	1609	RBC	$\nu(C_a = C_b)$, $\nu(C = C)_{vinyl}$[73]
1096	*S. epi*	$\nu_s(PO_2)$[76]	1614	*E. coli*	adenine, guanine (ring stretching)[69]

Peak Pos. (cm^{-1})	Cell Type	Peak Assignment	Peak Pos. (cm^{-1})	Cell Type	Peak Assignment
1097	*E. coli*	$\nu_s(PO_2)$[76]	1617	RBC	C=C trp, tyrosine[75]
1099	*E. coli*	carbohydrates, C-C, C-O, -C-OH[69]	1620	RBC	$\nu(C = C)_{vinyl}$[74]
1109	RBC	Proteins, lipids: C–N and C–C stretch[73]	1635	RBC	$\nu(C_\alpha C_m)_{asym}$, $\nu 10$[73]
1120	RBC	$\nu 5$[74]			

Note: The Raman shift can vary slightly, depending on cell culture media, bacterial strain, and Raman substrate as well as acquisition parameters including excitation wavelength and temperature[73], and as such, the peak assignments are often treated as approximations[67].

Abbreviations: pyr, pyrrol; deform, deformation; sym, symmetric; asym, asymmetric; Cys, cysteine; Lys, lysine; Glu, glutamic acid; Ile, isoleucine; Phe, phenylalanine; Met, methionine; His, histidine; Asp, aspartic acid; Asn, asparagine; Gln, glutamine; Ala, alanine; Leu, leucine; Val, valine; Trp, tryptophan; Ser, serine; Thr, threonine; breathe, breathing; sciss, scissoring; stretch, stretching; wag, wagging. Vibrations: ν, valence; δ, deformation, γ, deformation (out of plane)[73].

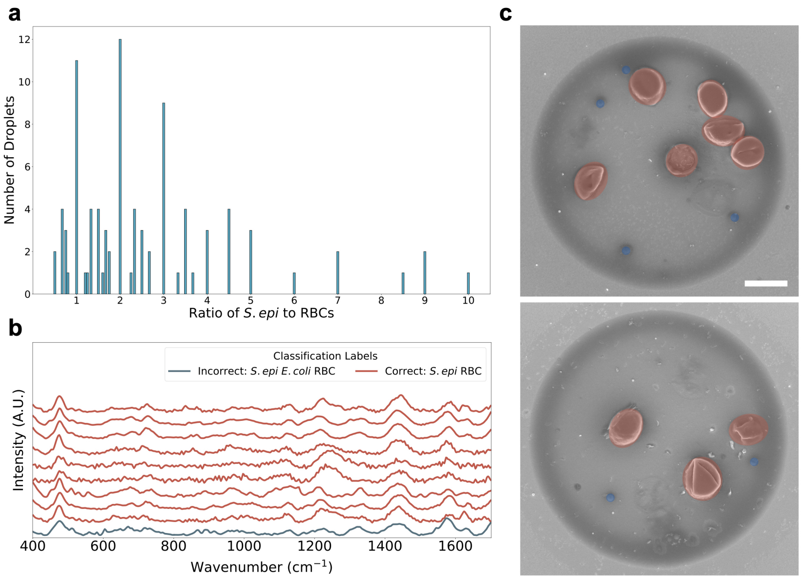

Supplementary Fig. 27 We further analyze samples printed from mixtures of *S. epi* bacteria, RBCs, and GNRs to understand how our ML algorithms would handle droplets printed with a greater number of RBCs as compared to *S. epi*. For the 100 droplets analyzed in Figure 4 from the main text, we count the number of *S. epi* bacteria and RBCs and **a,** plot ratio of *S. epi*:RBCs in each of the droplets. Of note, 8 droplets had 0 RBCs and therefore are not included on the plot. **b,** There were 10 droplets with a greater number of RBCs than *S. epi* in each droplet. For these droplets, we train our ML algorithm on the remaining 590 spectra in our 6 cellular classes. We then test it on these 10 spectra. The pre-processed spectra are plotted above, with the color indicating the classifier prediction. We got a 90% classification accuracy, with 1 spectra falsely classified as containing both *S. epi* and *E. coli* bacteria along with RBCs. **c,** We show two representative SEMs from these 10 droplets, with the RBCs and *S. epi* false colored in red and blue, respectively. The scale bar is 5 μm. These results are indicative of what we might see in a more clinically relevant sample with a greater concentration of RBCs than bacteria. We see that even in our limited dataset, we get comparable classification accuracies to samples with greater bacterial numbers, showing promise for our platform's ability to detect lower bacterial concentrations.

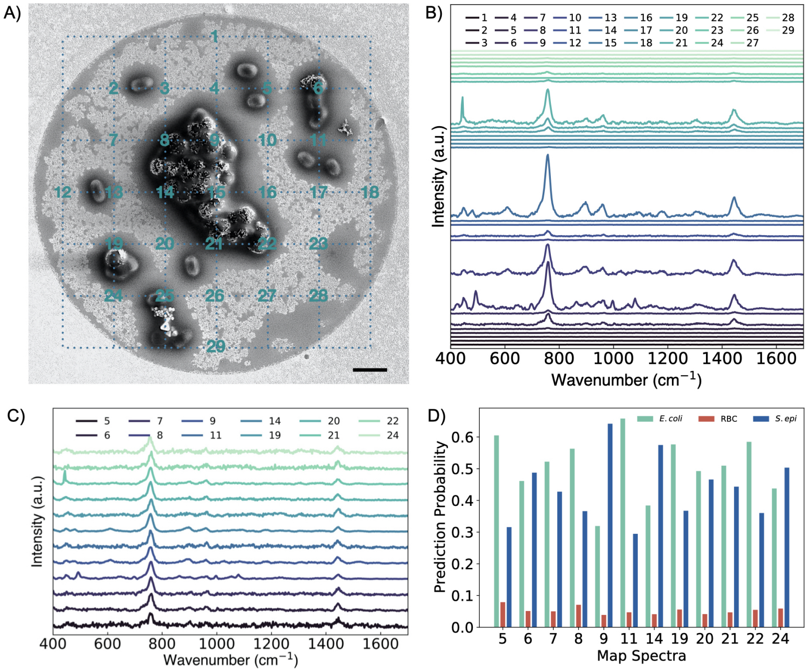

Supplementary Fig. 28 Raman mapping of droplets printed from *S. epi*, *E. coli*, and GNR mixtures with a focal spot size of 0.7 μm chose to allow for single cell interrogation. **a,** SEM of a single droplet with an overlay showing the 29 XY coordinates of spectra collection points. Scale bar is 2 μm. **b,** Waterfall plot showing the Raman spectra collected at each XY location. Spectra were preprocessed with a baseline fit. **c,** Waterfall plot showing the 12 remaining spectra after further preprocessing. First, spectra below an intensity count of 150 were removed to eliminate spectra taken from the substrate in locations without cells. Subsequently, remaining spectra were normalized to a zero mean and unit variance using the Scikit-learn python library Standard Scaler function [16]. **d,** Processed spectra were then evaluated using a SVM, Scikit-learn,[16] optimized and trained on our known sample set of 300 droplets (Figure 3c). The plot shows the prediction probabilities across each of our 3 cell types, predicted for each processed spectrum.

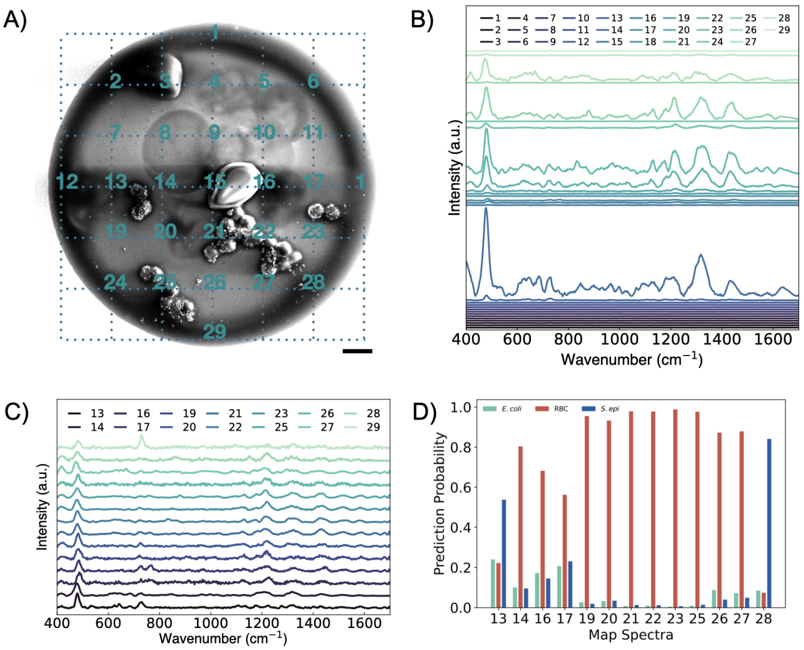

Supplementary Fig. 29 Raman mapping of droplets printed from *S. epi*, RBCs, and GNR mixtures with a focal spot size of 0.7 μm chose to allow for single cell interrogation. **a,** SEM of a single droplet with an overlay showing the 29 XY coordinates of spectra collection points. Scale bar is 2 μm. **b,** Waterfall plot showing the Raman spectra collected at each XY location. Spectra were preprocessed with a baseline fit. **c,** Waterfall plot showing the 14 remaining spectra after further preprocessing. First, spectra below an intensity count of 150 were removed to eliminate spectra taken from the substrate in locations without cells. Subsequently, remaining spectra were normalized to a zero mean and unit variance using the Scikit-learn python library Standard Scaler function [16].**d,** Processed spectra were then evaluated using a SVM, Scikit-learn,[16] optimized and trained on our known sample set of 300 droplets (Figure 3c). The plot shows the prediction probabilities across each of our 3 cell types, predicted for each processed spectrum.

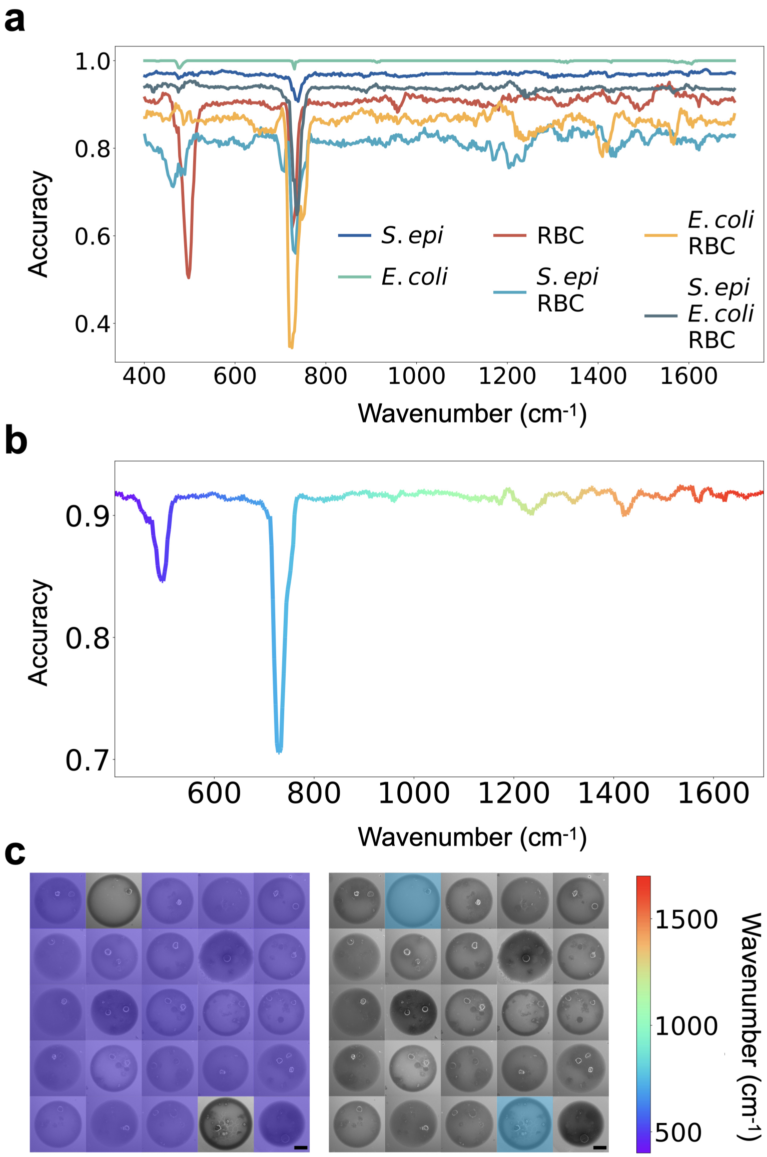

Supplementary Fig. 30 Future vision for Raman hyperspectral imaging. **a,** Calculated plot of the classification accuracy for each independent sample class, highlighting wavenumber importance for each individual class. Wavenumbers with lower accuracies are shown to be critical features as random perturbations are highly correlated with decreases in classification accuracy. **b,**Calculated plot of the mean classification accuracy across all 6 sample classes, calculated across all perturbation trials. Plot is shown as a rainbow spectrum to correspond with simulated hyperspectral images shown in **c**. Plot is identical to that shown in the main text Fig. 4d. **c,** Simulated image showing a grid array of 25 droplet SEM. 23 droplets contain only RBCs and GNRs while 2 droplets contain a mix of *S. epi* bacteria, RBCs, and GNRS. The image on the left shows the droplets containing only RBCs lighting up when interrogated with 816 nm light, while the image on the right shows droplets containing a mixture of bacteria and RBCs lighting up when interrogated with light at 833 nm. Chosen wavelengths correspond to accuracy dips associated with each droplet class. Scale bar is 10 μm.

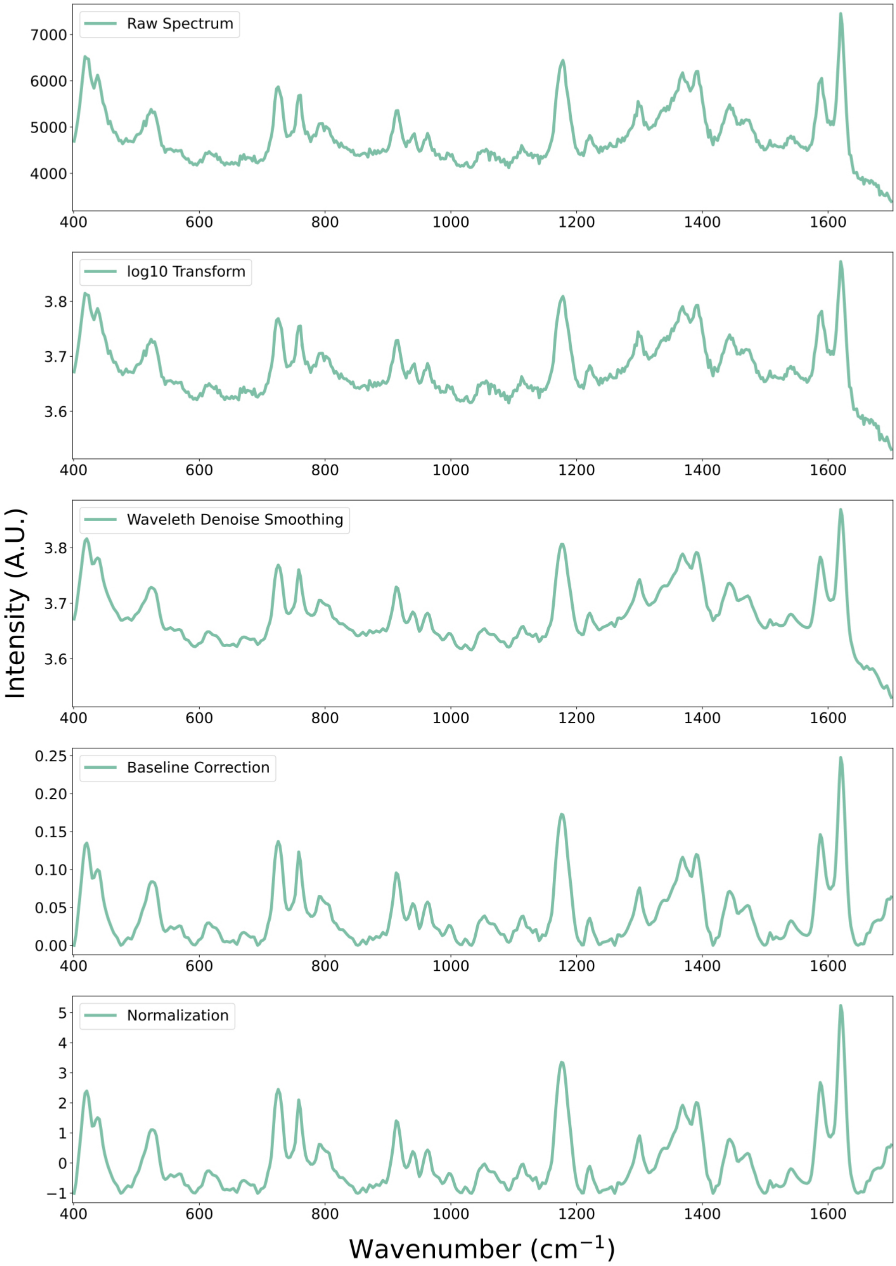

Supplementary Fig. 31 Spectral Preprocessing. Plot showing a sample spectra taken from our dataset of spectra collected from droplets printed with *E. coli* bacteria and GNRs. Plot shows (from top to bottom) the raw spectrum, the spectrum after a $\log_{10}$ transformation, spectrum after smoothing using a wavelet denoising, spectrum with baseline correction, and finally the normalized spectrum.

References

[1] Murray, C. B. Using binary surfactant mixtures to simultaneously improve the dimensional tunability and monodispersity in the seeded growth of gold nanorods. *Nano Lett.* **13** (2), 765–771 (2013) .

[2] Dionne, J. Plasmonic and electrostatic interactions enable uniformly enhanced liquid bacterial Surface-Enhanced raman scattering (SERS). *Nano Lett.* **20** (10), 7655–7661 (2020) .

[3] & Pradeep, T. Reversible assembly and disassembly of gold nanorods induced by EDTA and its application in SERS tuning. *Langmuir* **27** (7), 3381–3390 (2011) .

[4] Pignataro, B. Inkjet printing ag nanoparticles for SERS hot spots. *Anal. Methods* **10** (26), 3215–3223 (2018) .

[5] & Moehwald, H. Prospects for plasmonic hot spots in single molecule SERS towards the chemical imaging of live cells. *Phys. Chem. Chem. Phys.* **17** (33), 21072–21093 (2015) .

[6] Song, Y. Inkjet printing patterned photonic crystal domes for wide viewing-angle displays by controlling the sliding three phase contact line. *Adv. Opt. Mater.* **2** (1), 34–38 (2014) .

[7] Song, Y. Highly reproducible SERS arrays directly written by inkjet printing. *Nanoscale* **7** (2), 421–425 (2014) .

[8] Li, Y. Spherical nanoparticle arrays with tunable nanogaps and their hydrophobicity enhanced rapid SERS detection by localized concentration of droplet evaporation. *Adv. Mater. Interfaces* **2** (9), 1500031 (2015) .

[9] Yin, P. Superhydrophobic 3D forest-like ag microball/nanodendrite hierarchical structure as SERS sensor for rapid droplets detection. *Adv. Mater. Interfaces* **6** (8), 1801966 (2019) .

[10] Lee, T. A droplet-based high-throughput SERS platform on a droplet-guiding-track-engraved superhydrophobic substrate. *Small* **13** (7), 1602865 (2017) .

[11] Panigrahi, S. Application of vegetation indices for agricultural crop yield prediction using neural network techniques. *Remote Sensing* **2**, 673–696 (2010) .

[12] Mahato, K. K. Exploring photoacoustic spectroscopy-based machine learning together with metabolomics to assess breast tumor progression in a xenograft model ex vivo. *Lab. Invest.* **101** (7), 952–965 (2021) .

[13] & Sümmchen, L. Spike removal and denoising of raman spectra by wavelet transform methods. *Anal. Chem.* **73** (17), 4364–4373 (2001) .

[14] & Ruisánchez, I. Noise and background removal in raman spectra of ancient pigments using wavelet transform. *J. Raman Spectrosc.* **36** (9), 848–856 (2005) .

[15] Oliphant, T. E. Array programming with NumPy. *Nature* **585** (7825), 357–362 (2020) .

[16] Duchesnay, É. Scikit-learn: Machine learning in python. *J. Mach. Learn. Res.* **12** (85), 2825–2830 (2011) .

[17] & Marquardt, W. Automatic generation of peak-shaped models. *Appl. Spectrosc.* **58** (8), 986–994 (2004) .

[18] Sundius, T. Computer fitting of voigt profiles to raman lines. *J. Raman Spectrosc.* **1** (5), 471–488 (1973) .

[19] Fabris, L. Gold nanostar substrates for SERS-based chemical sensing in the femtomolar regime. *Nanoscale* **6** (15), 8891–8899 (2014) .

[20] & Halas, N. J. Surface-enhanced raman scattering on tunable plasmonic nanoparticle substrates. *Proc. Natl. Acad. Sci. U. S. A.* **101** (52), 17930–17935 (2004) .

[21] Hillenbrand, R. Resolving the electromagnetic mechanism of surface-enhanced light scattering at single hot spots. *Nat. Commun.* **3** (1), 1–7 (2012) .

[22] Liz-Marzán, L. M. Present and future of Surface-Enhanced raman scattering. *ACS Nano* **14** (1), 28–117 (2020) .

[23] Aussenegg, F. R. Optimized surface-enhanced raman scattering on gold nanoparticle arrays. *Appl. Phys. Lett.* **82** (18), 3095–3097 (2003) .

[24] & Birke, R. L. The theory of surface-enhanced raman scattering. *J. Chem. Phys.* **136** (14), 144704 (2012) .

[25] Kircher, M. F. Surface-Enhanced raman spectroscopy: A new modality for cancer imaging. *J. Nucl. Med.* **56** (9), 1295–1299 (2015) .

[26] Fabris, L. A review on Surface-Enhanced raman scattering. *Biosensors* **9** (2) (2019) .

[27] Van Duyne, R. P. (eds Kneipp, H.) *Surface-Enhanced Raman Scattering: Physics and Applications* 19–45 (Springer Berlin Heidelberg, Berlin, Heidelberg, 2006).

[28] Deckert, V. Spatial resolution of tip-enhanced raman spectroscopy - DFT assessment of the chemical effect. *Nanoscale* **8** (19), 10229–10239 (2016) .

[29] Haynes, C. L. Recent progress in SERS biosensing. *Phys. Chem. Chem. Phys.* **13** (24), 11551–11567 (2011) .

[30] Murphy, C. J. Aspect ratio dependence on surface enhanced raman scattering using silver and gold nanorod substrates. *Phys. Chem. Chem. Phys.* **8** (1), 165–170 (2006) .

[31] Ray, P. C. Gold nanoparticle based Label-Free SERS probe for ultrasensitive and selective detection of trinitrotoluene. *J. Am. Chem. Soc.* **131** (38), 13806–13812 (2009) .

[32] Kneipp, J. SERS enhancement of gold nanospheres of defined size. *J. Raman Spectrosc.* **42** (9), 1736–1742 (2011) .

[33] Odom, T. W. Nanoparticle SERS substrates with 3D raman-active volumes. *Chem. Sci.* **2** (8), 1435–1439 (2011) .

[34] Nam, J.-M. Multicomponent plasmonic nanoparticles: From heterostructured nanoparticles to colloidal composite nanostructures. *Chem. Rev.* **119** (24), 12208–12278 (2019) .

[35] Van Duyne, R. P. SERS: Materials, applications, and the future. *Mater. Today* **15** (1), 16–25 (2012) .

[36] Fadeel, B. Toxicology of engineered nanomaterials: focus on biocompatibility, biodistribution and biodegradation. *Biochim. Biophys. Acta* **1810** (3), 361–373 (2011) .

[37] Sastry, M. Biocompatibility of gold nanoparticles and their endocytotic fate inside the cellular compartment: a microscopic overview. *Langmuir* **21** (23), 10644–10654 (2005) .

[38] Bardhan, R. PRADA: Portable reusable accurate diagnostics with nanostar antennas for multiplexed biomarker screening. *Bioeng. Transl. Med.* **5** (3), e10165 (2020) .

[39] Liz-Marzán, L. M. Anisotropic metal nanoparticles for surface enhanced raman scattering. *Chem. Soc. Rev.* **46** (13), 3866–3885 (2017) .

[40] Nafisah, S. Controlled wet chemical synthesis of gold nanorods for triclopyr butotyl herbicide detection based-plasmonic sensor. *Sensing and Bio-Sensing Research* **29**, 100359 (2020) .

[41] El-Sayed, M. A. Calculated absorption and scattering properties of gold nanoparticles of different size, shape, and composition: applications in

biological imaging and biomedicine. *J. Phys. Chem. B* **110** (14), 7238–7248 (2006) .

[42] Ren, B. Size effect on SERS of gold nanorods demonstrated via single nanoparticle spectroscopy. *J. Phys. Chem. C* **120** (37), 20806–20813 (2016) .

[43] Srinivasarao, M. Colloidal dispersion of gold nanorods: Historical background, optical properties, seed-mediated synthesis, shape separation and self-assembly. *Mater. Sci. Eng. R Rep.* **65** (1), 1–38 (2009) .

[44] Smith, D. R. Extreme nanophotonics from ultrathin metallic gaps. *Nat. Mater.* **18** (7), 668–678 (2019) .

[45] Wallace, P. M. Inverted size-dependence of surface-enhanced raman scattering on gold nanohole and nanodisk arrays. *Nano Lett.* **8** (7), 1923–1928 (2008) .

[46] Rowlen, K. L. Quantitative evaluation of plasmon enhanced raman scattering from nanoaperture arrays. *J. Phys. Chem. C* **111** (4), 1689–1694 (2007) .

[47] Loh, K. P. Large-Area graphene nanodot array for Plasmon-Enhanced infrared spectroscopy. *Small* **12** (10), 1302–1308 (2016) .

[48] Narayana, C. Hot spots in ag Core-Au shell nanoparticles potent for Surface-Enhanced raman scattering studies of biomolecules. *J. Phys. Chem. C* **111** (11), 4388–4392 (2007) .

[49] Qiu, C. W. Electromagnetic theory of tunable SERS manipulated with spherical anisotropy in coated nanoparticles. *J. Phys. Chem. C* **115** (18), 8893–8899 (2011) .

[50] Halas, N. J. Controlling the surface enhanced raman effect via the nanoshell geometry. *Appl. Phys. Lett.* **82** (2), 257–259 (2003) .

[51] Quate, C. F. IEEE (ed.) *Acoustic ink printing.* (ed.IEEE) *IEEE 1992 Ultrasonics Symposium Proceedings*, 929–935 vol.2 (1992).

[52] Lundgren, T. S. Nozzleless droplet formation with focused acoustic beams. *J. Appl. Phys.* **65** (9), 3441–3447 (1989) .

[53] & Apfel, R. E. Acoustic radiation pressure produced by a beam of sound. *J. Acoust. Soc. Am.* **72** (6), 1673–1687 (1982) .

[54] Rayleigh, J. W. *The Theory of Sound, 2nd Ed.* (Dover Publications, New York, 1945).

[55] Takayama, S. Rapid generation of multiplexed cell cocultures using acoustic droplet ejection followed by aqueous two-phase exclusion patterning. *Tissue Eng. Part C Methods* **18** (9), 647–657 (2012) .

[56] Ellson, R. Moving liquids with sound: The physics of acoustic droplet ejection for robust laboratory automation in life sciences. *J. Lab. Autom.* **21** (1), 4–18 (2016) .

[57] Eisenmenger, W. Dynamic properties of the surface tension of water and aqueous solutions of surface active agents with standing capillary waves in the frequency range from 10 kc/s to 1.5 mc/s. *Acta Acustica united with Acustica* **9** (4), 327–340 (1959) .

[58] Sprague, R. & Schneider, S. (eds) (eds *Acoustic ink printing: an application of ultrasonics for photographic quality printing at high speed* & Schneider, S.) *2001 IEEE Ultrasonics Symposium. Proceedings. An International Symposium (Cat. No.01CH37263)*, Vol. 1, 627–635 vol.1 (2001).

[59] Zhou, Q. Three-dimensional bio-printing. *Sci. China Life Sci.* **58** (5), 411–419 (2015) .

[60] & Montesano, G. Single cell epitaxy by acoustic picolitre droplets. *Lab Chip* **7** (9), 1139–1145 (2007) .

[61] Zouni, A. Acoustic injectors for Drop-On-Demand serial femtosecond crystallography. *Structure* **24** (4), 631–640 (2016) .

[62] Yano, J. Drop-on-demand sample delivery for studying biocatalysts in action at x-ray free-electron lasers. *Nat. Methods* **14** (4), 443–449 (2017) .

[63] Orville, A. M. Acoustically mounted microcrystals yield high-resolution x-ray structures. *Biochemistry* **50** (21), 4399–4401 (2011) .

[64] Bostwick, J. R. Acoustic droplet ejection applications for High-Throughput screening of infectious agents. *J. Lab. Autom.* **21** (1), 188–197 (2016) .

[65] Caprioli, R. M. Automated acoustic matrix deposition for MALDI sample preparation. *Anal. Chem.* **78** (3), 827–834 (2006) .

[66] Zhong, R.-G. Rapid detection of escherichia coli and salmonella typhimurium by surface-enhanced raman scattering. *Optoelectronics Letters* **11**, 157–160 (2015) .

[67] Piskin, E. Bacterial detection using bacteriophages and gold nanorods by following time-dependent changes in raman spectral signals. *Artif. Cells*

Nanomed. Biotechnol. **46** (sup2), 122–130 (2018) .

[68] Kamińska, A. Sources of variability in SERS spectra of bacteria: comprehensive analysis of interactions between selected bacteria and plasmonic nanostructures. *Anal. Bioanal. Chem.* **411** (10), 2001–2017 (2019) .

[69] Irudayaraj, J. Silver nanosphere SERS probes for sensitive identification of pathogens. *J. Phys. Chem. C* **114** (39), 16122–16128 (2010) .

[70] Waluk, J. Nanostructured silver–gold bimetallic SERS substrates for selective identification of bacteria in human blood. *Analyst* **139** (5), 1037–1043 (2014) .

[71] Jung, J. H. Fully integrated optofluidic SERS platform for real-time and continuous characterization of airborne microorganisms **169**, 112611 (2020) .

[72] Haisch, C. SERS detection of bacteria in water by in situ coating with ag nanoparticles. *Anal. Chem.* **86** (3), 1525–1533 (2014) .

[73] Kneipp, J. SERS reveals the specific interaction of silver and gold nanoparticles with hemoglobin and red blood cell components. *Phys. Chem. Chem. Phys.* **15** (15), 5364–5373 (2013) .

[74] Ziegler, L. D. Surface-enhanced raman scattering of whole human blood, blood plasma, and red blood cells: cellular processes and bioanalytical sensing. *J. Phys. Chem. B* **116** (31), 9376–9386 (2012) .

[75] Bamrungsap, S. A simple paper-based surface enhanced raman scattering (SERS) platform and magnetic separation for cancer screening. *Sens. Actuators B Chem.* **285**, 462–469 (2019) .

[76] Pezzotti, G. Label-Free SERS discrimination and in situ analysis of life cycle in escherichia coli and staphylococcus epidermidis. *Biosensors* **8** (4) (2018) .
